# Benzamides Substituted with Quinoline-Linked 1,2,4-Oxadiazole: Synthesis, Biological Activity and Toxicity to Zebrafish Embryo

**DOI:** 10.3390/molecules27123946

**Published:** 2022-06-20

**Authors:** Bin-Long Sun, Ying-Ying Wang, Sen Yang, Min-Ting Tu, Ying-Ying Shao, Yi Hua, Yi Zhou, Cheng-Xia Tan

**Affiliations:** College of Chemical Engineering, Zhejiang University of Technology, Hangzhou 310014, China; zark725@163.com (B.-L.S.); wyy1252909407@163.com (Y.-Y.W.); y17764528124@163.com (S.Y.); tt1239803789@163.com (M.-T.T.); s2901692009@163.com (Y.-Y.S.); huayi3181@gmail.com (Y.H.); 2112101265@zjut.edu.cn (Y.Z.)

**Keywords:** quinoline, synthesis, 1,2,4-oxadiazole, biological activity, toxicity

## Abstract

To develop new compounds with high activity, broad spectrum and low-toxicity, 17 benzamides substituted with quinoline-linked 1,2,4-oxadiazole were designed using the splicing principle of active substructures and were synthesized. The biological activities were evaluated against 10 fungi, indicating that some of the synthetic compounds showed excellent fungicidal activities. For example, at 50 mg/L, the inhibitory activity of **13p** (3-Cl-4-Cl substituted, 86.1%) against *Sclerotinia sclerotiorum* was superior to that of quinoxyfen (77.8%), and the inhibitory activity of **13f** (3-CF_3_ substituted, 77.8%) was comparable to that of quinoxyfen. The fungicidal activities of **13f** and **13p** to *Sclerotinia sclerotiorum* were better than that of quinoxyfen (14.19 mg/L), with EC_50_ of 6.67 mg/L and 5.17 mg/L, respectively. Furthermore, the acute toxicity of **13p** was 19.42 mg/L, classifying it as a low-toxic compound.

## 1. Introduction

**Figure 1:**
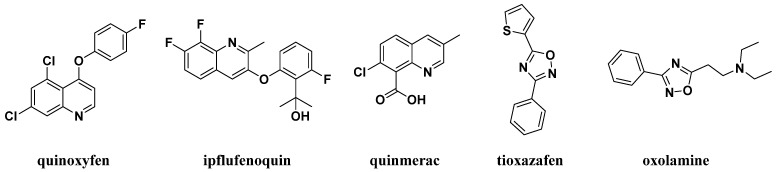
Chemical structures of quinoxyfen, ipflufenoquin, quinmerac, tioxazafen and oxolamine.

Possessing different biological activity, heterocyclic structures like nitrogen-containing heterocyclic [1,2] and oxygen-containing heterocyclic are important features in synthetic pesticides for their high efficiency, various biological activities, and diversity of possible substituents. Quinoline is a versatile group, a privileged scaffold, and an outstanding fused heterocyclic compound [3]. Apart from their applications in medicine [4], quinoline derivatives have shown potential in pesticides, such as insecticidal [5,6,7], herbicidal [8] and fungicidal activities [9,10,11,12]. As the bioisostere of the amide, the 1,2,4-oxadiazole heterocycle has good hydrolytic and metabolic properties [13], and exhibits a wide range of biological activities in the field of pesticides [14,15,16,17,18,19]. In addition, there have already been quite a few products containing quinoline or 1,2,4-oxadiazole scaffold launched successively, including quinoxyfen [20], ipflufenoquin [21], quinmerac, tioxazafen and oxolamine (Figure 1).

**Figure 2:**
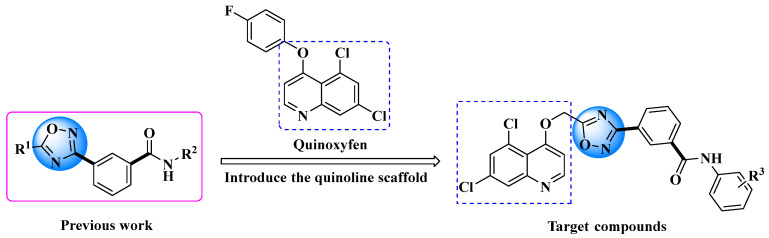
Design strategy of the target compounds.

In our efforts to develop potent fungicides, we have previously reported the synthesis and biological activity studies of 1,2,4-oxadiazole-substituted benzamide derivatives [22,23]. Some of them exhibited good fungicidal activities. In view of the facts mentioned above, to further improve the fungicidal activities of these compounds, we designed (Figure 2) a series of novel 1,2,4-oxadiazole-substituted benzamides using the splicing principle of active substructures and synthesized them by introducing a quinoline scaffold at the 5-position of 1,2,4-oxadiazole. The chemical structures of these new compounds were confirmed by ^1^H-NMR, ^13^C-NMR, and HRMS, their fungicidal activities were studied and a toxicity test with zebrafish embryo was performed.

## 2. Results and Discussion

### 2.1. Synthesis of Target Compounds

**Scheme 1:**
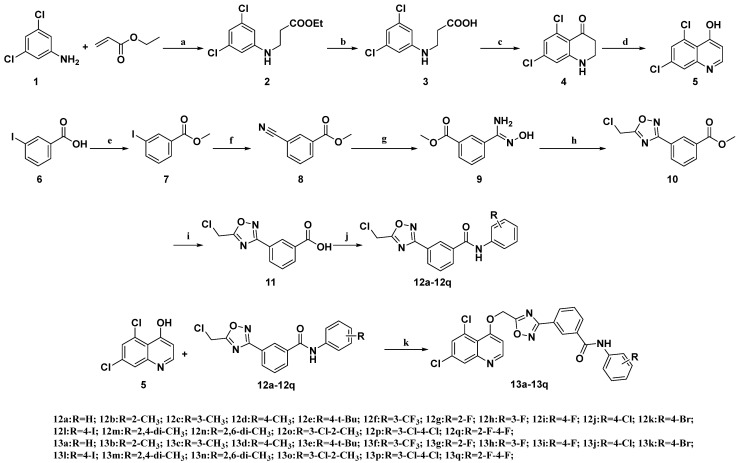
Synthetic route of target compounds. Reagents and conditions: (**a**) MSA, 60 °C; (**b**) CH_3_OH, OH^−^, 60 °C; (**c**) PPA, 150 °C; (**d**) MeCN, H_2_SO_4_, K_2_S_2_O_8_, reflux; (**e**) CH_3_OH, H^+^, reflux; (**f**) CuCN, l-proline, DMF, 100 °C; (**g**) NH_2_OH·HCl, CH_3_CH_2_OH, rt; (**h**) ClCH_2_COCl, Et_3_N, toluene, reflux; (**i**) CH_3_COOH, HCl, 70 °C; (**j**) SOCl_2_, reflux; THF, Et_3_N, 0 °C; (**k**) K_2_CO_3_, DMF, 60 °C.

The synthetic pathway to target compounds **13a**–**13q** is shown in Scheme 1. The starting material 3,5-dichloroaniline **1** underwent addition, hydrolysis, cyclization, and oxidation reaction to give 5,7-dichloro-4-hydroxyquinoline **5**. During the addition reaction (the first reaction in Scheme 1), methanesulfonic acid (MSA) was selected as the acid catalyst and the optimum molar ratio of 1:0.2 was determined, which greatly improved the yield of the reaction. In step 4, we performed a preliminary screening of the oxidant, and finally determined to take the K_2_S_2_O_8_/H_2_SO_4_ system as the oxidant to afford compound **5** in the best yield. The influence of different reaction conditions on the yield of the compound **5** is shown in Table 1. Moreover, acetonitrile was chosen as the solvent in which the product had low solubility so that it could precipitate to obtain the solid easily.

**Scheme 2:**
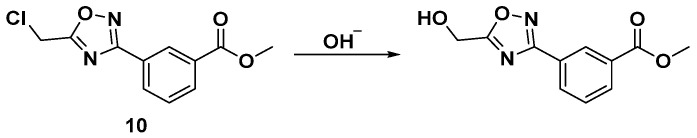
Hydrolysis reaction of compound **10** under alkaline condition.

The synthesis of amine oxime **9** was similar to our previous procedures [23]. It should be noted that this reaction could not be carried out for a long time to avoid the form of amide by-products. Afterwards, intermediate **12** was prepared from compound **9** via cyclization, hydrolysis, and condensation reaction. In addition, the hydrolysis reaction was carried out under acidic condition to avoid by-products as chlorine-substituted alkanes would hydrolyzed readily than ester groups under alkaline condition (Scheme 2).

Finally, Williamson ether synthesis of compound **5** with **12** formed the target compounds **13**.

### 2.2. Spectrum Analysis of Target Compounds

All the target compounds were confirmed by ^1^H-NMR, ^13^C-NMR, and HRMS. The target compound **13f** was taken as an example to conduct spectrum analysis. In the ^1^H-NMR spectra of **13f**, the –NH– proton signal was found at δ 10.75 ppm. In addition, the single peak at 6.04 ppm was the peak of –CH_2_– between ether bond and 1,2,4-oxadiazoles. In the ^13^C-NMR spectra of compound **13f**, the appearances of signals at 167.83 ppm and 165.40 ppm were assigned to the carbons of the 1,2,4-oxadiazole ring. In the HRMS spectrogram, the calculated value of the ion peak of this compound was [M + Na]^+^ 559.0546, and the measured value was [M + Na]^+^ 559.0549. The absolute error was within 0.003.

### 2.3. Biological Activities of Target Compounds

The results of the fungicidal activities test of the target compounds against 10 fungi are shown in Table 2. At 50 mg/L, all the target compounds **13a**–**13q** were found to exhibit certain inhibitory activity against the 10 fungi tested. Overall, the target compounds showed better inhibitory activity against *Sclerotinia sclerotiorum*, ranging from 47.2% to 86.1%. Among them, the inhibitory rate of compound **13p** (86.1%) was superior to the control drug quinoxyfen (77.8%), and the inhibitory rate of compound **13f** was 77.8%, which was similar to quinoxyfen. In addition, the inhibition rates of compounds **13a**, **13b**, **13d** and **13o** against *Sclerotinia sclerotiorum* were 75.0%, 72.2%, 75.0% and 75.0%, respectively, which are slightly lower than that of quinoxyfen. Other compounds also exhibited moderate inhibitory activity (47.2–69.4%). For *Alternaria solani, Gibberella zeae, Phytophthora capsica* and *Physalospora piricola*, some compounds possessed better inhibitory activities than quinoxyfen, but their inhibition rates were less than 50%. As can be seen from Table 3, the EC_50_ of compounds **13f** and **13p** against *Sclerotinia sclerotiorum* were 6.67 mg/L and 5.17 mg/L, respectively, which were significantly superior to quinoxyfen (14.19 mg/L). Structure–activity relationship (SAR) results for these target compounds showed that when the substituent of the benzene ring was 3-CF_3_ or 3,4-(Cl)_2_, their inhibitory activities were obviously superior to others. Overall, electron withdrawing groups are beneficial to inhibitory activity.

### 2.4. Toxicity to Zebrafish Embryo

**Figure 3:**
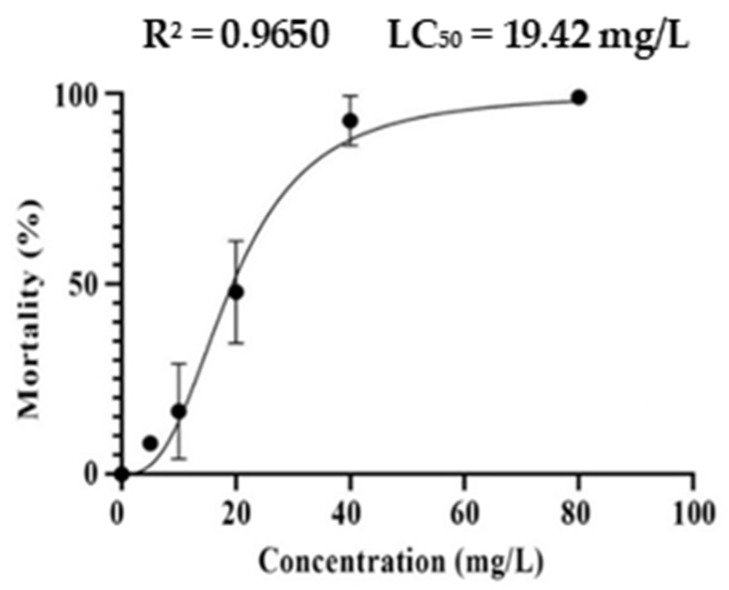
Zebrafish embryo mortality rates after exposure to **13p**.

According to the fungicidal activity results (Figure 3), we selected compound **13p** with better activity to study the lethal and teratogenic effects exposure on zebrafish embryos from 6 to 96 hpf (hours post fertilization). When the concentration of **13p** was below 40 mg/L, the mortality rate increased sharply as the concentration increased. Afterwards, the mortality rate exceeded 90% at 40 mg/L. The resulting LC_50_ value for compound **13p** was 19.42 mg/L, and it was classified as a low-toxic compound [24].

**Figure 4:**
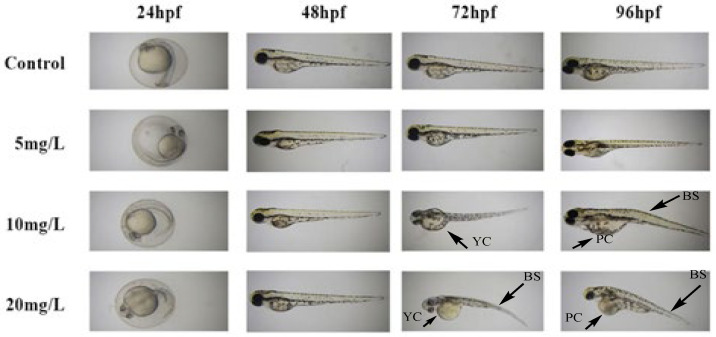
Zebrafish embryo malformation after exposure to compound **13p**. Note: BS, bent spine; PC, pericardial cyst; YC, yolk cyst.

As the time and concentration increased, zebrafish embryos showed obvious developmental delay (Figure 4), such as bent spine, pericardial cyst, yolk cyst and even malformation. At 72 hpf, compared to the control group, the zebrafish embryo exposed at 10 mg/L and 20 mg/L showed obvious yolk cyst. At 96 hpf, pericardial cyst and bent spine appeared on the zebrafish embryo exposed at 10 mg/L and 20 mg/L.

## 3. Experimental Section

### 3.1. General Information

Melting points were determined using an X-4 digital microscopic melting point detector (Taike, Beijing, China) and the thermometer was uncorrected. ^1^H-NMR and ^13^C-NMR spectra were measured on BRUKER Avance 500 MHz spectrometer (Bruker 500 MHz, Fallanden, Switzerland) using CDCl_3_ or DMSO as the solvent. High-resolution electrospray mass spectra (HR-ESI–MS) were determined using an UPLC H CLASS/QTOF G2 XS mass spectrometer (Waters, Milford, CT, USA). All the reagents were analytical grade or synthesized in our laboratory. The characterization data for all synthetic compounds are provided in the Supplementary Materials.

Ethics statement: The Institutional Animal Care and Use Committee (IACUC) at Wenzhou Medical University (SYXK 2019-0009, 4 April 2019 to 4 April 2024) approved our study plan for proper use of zebrafish. All studies were carried out in strict accordance with the guidelines of the IACUC. All dissections were performed on ice, and all efforts were made to minimize suffering.

### 3.2. Synthesis

#### 3.2.1. Ethyl 3-((3,5-dichlorophenyl)amino)propanoate (**2**)

3,5-dichloroaniline **1** (16.20 g, 0.10 mol) and ethyl acrylate (30.00 g, 0.30 mol) were sequentially added to a three-necked flask, heated, and stirred until dissolved completely. The mixture of MSA (1.44 g) and water (2.70 g) was added dropwise, then reacted at 60 °C for 16 h. After the reaction was completed, the mixture was cooled to room temperature, unreacted ethyl acrylate was removed under reduced pressure. The remnant was dissolved in toluene (300 mL) and washed with HCl. Finally, the organic layer was dried with anhydrous MgSO_4_ and evaporated to give 23.60 g yellow solid. Yield: 90.0%, m.p. 72–74 °C; ^1^H-NMR (500 MHz, Chloroform-*d*) *δ* 6.69 (t, *J* = 1.7 Hz, 1H), 6.48 (d, *J* = 1.8 Hz, 2H), 4.18 (q, *J* = 7.1 Hz, 2H), 3.42 (t, *J* = 6.2 Hz, 2H), 2.61 (t, *J* = 6.2 Hz, 2H), 1.29 (t, *J* = 7.1 Hz, 3H).

#### 3.2.2. 3-((3,5-Dichlorophenyl)amino)propanoic Acid (**3**)

Ethyl 3-((3,5-dichlorophenyl)amino)propanoate **2** (31.32 g, 0.12 mol), methanol (50 mL) and NaOH (20%, 32.00 g) were added to a three-necked flask and reacted at 60 °C for 1 h. Methanol was removed under reduced pressure followed by the addition of water (100 mL). Afterwards, we adjusted the pH to 2–3 with HCl and white solid precipitate was obtained (24.50 g). Yield: 85%, m.p. 102–103 °C.

#### 3.2.3. 5,7-Dichloro-2,3-dihydroquinolin-4(1*H*)-one (**4**)

To a three-necked flask, we added PPA (10.00 g) and heat at 90 °C for 0.5 h. Then, 3-((3,5-dichlorophenyl)amino)propanoic acid **3** (4.66 g, 0.02 mol) was added slowly and reacted at 150 °C for 5 h. To the stirred solution, water (100 mL) was added to precipitate yellow solid after the mixture was cooled to room temperature. The crude product was filtered, sequentially washed with petroleum ether and saturated aqueous NaHCO_3_ solution, and dried to obtain 4.30 g solid. Yield: 94%, m.p. 184–185 °C.

#### 3.2.4. 5,7-Dichloro-4-hydroxyquinoline (**5**)

Conc. H_2_SO_4_ (2.00 g) was added slowly to a solution of compound **4** (5.00 g, 23.00 mmol) in acetonitrile (35.00 g). Afterwards, K_2_S_2_O_8_ (8.00 g) was added when the temperature reached 50 °C. The mixture was then reflux for 4 h. TLC was used to track the reaction progress. After the reaction was completed, the mixture was cooled to room temperature to precipitate solid. The solid was filtered, washed with water, and dried to obtain product **5** (4.60 g). Yield: 93.4%.

#### 3.2.5. Methyl-3-(*N*-hydroxycarbamimidoyl)benzoate (**9**)

The synthesis of intermediate **9** was performed with reference to our previous work.

#### 3.2.6. Methyl 3-(5-(chloromethyl)-1,2,4-oxadiazol-3-yl)benzoate (**10**)

To a three-necked flask, we added intermediate **9** (0.97 g, 5.00 mmol), triethylamine (1.20 g, 12.00 mmol) and dry toluene (100 mL). Stirring was started at 0 °C for 2 h followed by the dropwise addition of chloroacetyl chloride (0.58 g, 5.20 mmol). This was then reacted at 0 °C for another 3 h. The mixture was further heated to reflux for about 2 h. The mixture was then cooled to room temperature and sequentially washed with water and saturated sodium chloride solution. The organic layer was dried with Na_2_SO_4_ and the solvent was removed to give 0.93 g yellow solid. Yield: 73.8%; ^1^H-NMR (500 MHz, Chloroform-*d*) *δ* 8.75 (s, 1H), 8.28 (d, *J* = 7.8 Hz, 1H), 8.20 (d, *J* = 7.8 Hz, 1H), 7.59 (t, *J* = 7.8 Hz, 1H), 4.78 (s, 2H), 3.97 (s, 3H).

#### 3.2.7. 3-(5-(Chloromethyl)-1,2,4-oxadiazol-3-yl)benzoic Acid (**11**)

Compound **10** (5.00 g, 0.02 mol), CH_3_COOH (30 mL), and HCl (30 mL) were added to a three-necked flask and reacted at 70 °C for 3 h. After the reaction was completed, the mixture was cooled to room temperature to precipitate white solid. The white solid was filtered, washed with water, and dried to give compound **11** (4.45 g). Yield: 93.6%, m.p. 179–182 °C; ^1^H NMR (400 MHz, DMSO-*d*_6_) *δ* 13.34 (s, 1H), 8.53 (s, 1H), 8.23 (d, *J* = 8.8 Hz, 1H), 8.14 (d, *J* = 7.8 Hz, 1H), 7.71 (t, *J* = 7.8 Hz, 1H), 5.19 (s, 2H).

#### 3.2.8. Synthesis of Intermediate (12)

The solution of compound **11** (0.24 g, 1.00 mmol) in SOCl_2_ (5 mL) was reacted at reflux for 3 h. The SOCl_2_ was removed and THF (30 mL) was added subsequently. Then, the mixture of substituted aniline (1.20 mmol), triethylamine (2.5 mmol) and THF (1 mL) was added dropwise under ice bath. Stirred overnight, separated by column chromatography to give intermediate **12**.

*3-(5-(chloromethyl)-1,2,4-oxadiazol-3-yl)-N-phenylbenzamide***12a**. Yellow solid, yield 79.4%, m.p. 103–104 °C; ^1^H-NMR (500 MHz, DMSO-*d*_6_) *δ* 10.52 (s, 1H), 8.60 (s, 1H), 8.22 (t, *J* = 9.0 Hz, 2H), 7.81 (d, *J* = 8.5 Hz, 2H), 7.76 (t, *J* = 7.8 Hz, 1H), 7.38 (t, *J* = 7.9 Hz, 2H), 7.13 (t, *J* = 7.4 Hz, 1H), 5.23 (s, 2H); ^13^C-NMR (126 MHz, DMSO-*d*_6_) *δ* 176.33, 168.18, 165.05, 139.41, 136.45, 131.42, 130.36, 130.06, 129.09, 126.82, 126.36, 124.37, 121.03, 34.21; HRMS calcd for C_16_H_12_ClN_3_O_2_ [M + H]^+^ 314.0691, found 314.0698.

*3-(5-(chloromethyl)-1,2,4-oxadiazol-3-yl)-N-(o-tolyl)benzamide***12b**. Yellow solid, yield 77.5%, m.p. 95–96 °C; ^1^H-NMR (500 MHz, DMSO-*d*_6_) *δ* 10.19 (s, 1H), 8.64 (t, *J* = 1.8 Hz, 1H), 8.24 (d, *J* = 7.7 Hz, 2H), 7.76 (t, *J* = 7.8 Hz, 1H), 7.36 (d, *J* = 7.4 Hz, 1H), 7.30 (d, *J* = 7.4 Hz, 1H), 7.27–7.17 (m, 2H), 5.23 (s, 2H), 2.26 (s, 3H); ^13^C-NMR (126 MHz, DMSO-*d*_6_) *δ* 176.32, 168.20, 164.91, 136.69, 136.07, 134.33, 131.31, 130.81, 130.32, 130.09, 127.17, 126.89, 126.64, 126.50, 126.41, 34.22, 18.39; HRMS calcd for C_17_H_15_ClN_3_O_2_ [M + H]^+^ 328.0847, found 328.0856.

*3-(5-(chloromethyl)-1,2,4-oxadiazol-3-yl)-N-(m-tolyl)benzamide***12c**. White solid, yield 69.7%, m.p. 98–100 °C; ^1^H-NMR (500 MHz, DMSO-*d*_6_) *δ* 10.43 (s, 1H), 8.60 (d, *J* = 1.8 Hz, 1H), 8.23 (d, *J* = 7.8 Hz, 1H), 8.20 (d, *J* = 7.9 Hz, 1H), 7.76 (t, *J* = 7.8 Hz, 1H), 7.64 (d, *J* = 1.8 Hz, 1H), 7.60 (d, *J* = 8.1 Hz, 1H), 7.26 (t, *J* = 7.8 Hz, 1H), 6.95 (d, *J* = 7.5 Hz, 1H), 5.23 (s, 2H), 2.33 (s, 3H); ^13^C-NMR (126 MHz, DMSO-*d*_6_) *δ* 176.32, 168.18, 164.96, 139.33, 138.25, 136.49, 131.40, 130.32, 130.05, 128.92, 126.78, 126.35, 125.06, 121.55, 118.20, 34.21, 21.66; HRMS calcd for C_17_H_15_ClN_3_O_2_ [M + H]^+^ 328.0847, found 328.0857.

*3-(5-(chloromethyl)-1,2,4-oxadiazol-3-yl)-N-(p-tolyl)benzamide***12d**. White solid, yield 73.8%, m.p. 113–116 °C; ^1^H-NMR (500 MHz, DMSO-*d*_6_) *δ* 10.43 (s, 1H), 8.59 (s, 1H), 8.24–8.18 (m, 2H), 7.75 (t, *J* = 7.8 Hz, 1H), 7.69 (d, *J* = 8.4 Hz, 2H), 7.18 (d, *J* = 8.2 Hz, 2H), 5.23 (s, 2H), 2.29 (s, 3H); ^13^C-NMR (126 MHz, DMSO-*d*_6_) *δ* 176.31, 168.19, 164.82, 136.89, 136.51, 133.35, 131.36, 130.26, 130.02, 129.47, 126.78, 126.34, 121.04, 34.21, 20.97; HRMS calcd for C_17_H_15_ClN_3_O_2_ [M + H]^+^ 328.0847, found 328.0854.

*3-(5-(chloromethyl)-1,2,4-oxadiazol-3-yl)-N-(4-(tert-butyl)phenyl)benzamide***12e**. White solid, yield 75.7%, m.p. 125–127 °C; ^1^H-NMR (500 MHz, DMSO-*d*_6_) *δ* 8.49 (s, 1H), 8.21 (d, *J* = 7.7 Hz, 1H), 8.15 (s, 1H), 8.05 (d, *J* = 7.7 Hz, 1H), 7.64–7.54 (m, 3H), 7.39 (d, *J* = 8.3 Hz, 2H), 4.76 (s, 2H), 1.34 (s, 9H); ^13^C-NMR (126 MHz, DMSO-*d*_6_) *δ* 167.72, 167.21, 164.88, 150.40, 146.74, 136.83, 136.53, 131.31, 130.51, 130.01, 126.94, 126.34, 125.71, 124.92, 120.74, 111.70, 41.05, 34.55, 31.67, 19.01, 12.96; HRMS calcd for C_20_H_21_ClN_3_O_2_ [M + H]^+^ 370.1317, found 370.1327.

*3-(5-(chloromethyl)-1,2,4-oxadiazol-3-yl)-N-(3-(trifluoromethyl)phenyl)benzamide***12f**. Yellow solid, yield 66.4%, m.p. 141–145 °C; ^1^H-NMR (500 MHz, DMSO-*d*_6_) *δ* 10.80 (s, 1H), 8.63 (d, *J* = 1.8 Hz, 1H), 8.31–8.17 (m, 3H), 8.10 (d, *J* = 8.1 Hz, 1H), 7.78 (t, *J* = 7.8 Hz, 1H), 7.63 (t, *J* = 8.0 Hz, 1H), 7.49 (d, *J* = 7.8 Hz, 1H), 5.23 (s, 2H); ^13^C-NMR (126 MHz, DMSO-*d*_6_) *δ* 167.60, 167.19, 165.40, 150.37, 140.21, 135.87, 131.37, 130.86, 130.33, 130.02 (d, *J* = 11.5 Hz), 126.97, 126.43, 125.69, 124.85, 124.38, 123.52, 120.63 (d, *J* = 3.8 Hz), 116.99 (d, *J* = 4.0 Hz), 111.72, 41.03, 18.98, 12.90; HRMS calcd for C_17_H_12_ClF_3_N_3_O_2_ [M + H]^+^ 382.0565, found 382.0576.

*3-(5-(chloromethyl)-1,2,4-oxadiazol-3-yl)-N-(2-fluorophenyl)benzamide***12g**. Yellow solid, yield 75.9%, m.p. 107–109 °C; ^1^H-NMR (500 MHz, DMSO-*d*_6_) *δ* 8.56 (s, 1H), 8.41 (t, *J* = 8.0 Hz, 1H), 8.27 (d, *J* = 7.8 Hz, 1H), 8.18 (s, 1H), 8.07 (d, *J* = 7.8 Hz, 1H), 7.64 (t, *J* = 7.8 Hz, 1H), 7.22–7.17 (m, 1H), 7.16–7.09 (m, 2H), 4.78 (s, 2H); ^13^C-NMR (126 MHz, DMSO-*d*_6_) *δ* 167.65, 167.19, 165.05, 157.29, 155.32, 150.38, 135.43, 131.45, 130.82, 130.10, 127.63 (d, *J* = 10.1 Hz), 127.06, 126.40, 125.98 (d, *J* = 12.2 Hz), 124.87, 124.79 (d, *J* = 3.5 Hz), 116.33 (d, *J* = 19.9 Hz), 111.71, 41.03, 19.00, 12.92; HRMS calcd for C_16_H_12_ClFN_3_O_2_ [M + H]^+^ 332.0597, found 332.0606.

*3-(5-(chloromethyl)-1,2,4-oxadiazol-3-yl)-N-(3-fluorophenyl)benzamide***12h**. Yellow solid, yield 78.6%, m.p. 123–127 °C; ^1^H-NMR (500 MHz, DMSO-*d*_6_) *δ* 10.68 (s, 1H), 8.59 (t, *J* = 1.5 Hz, 1H), 8.24 (d, *J* = 7.8 Hz, 1H), 8.20 (d, *J* = 8.4 Hz, 1H), 7.82–7.74 (m, 2H), 7.60 (d, *J* = 8.2 Hz, 1H), 7.45–7.37 (m, 1H), 6.97 (t, *J* = 8.5 Hz, 1H), 5.23 (s, 2H); ^13^C-NMR (126 MHz, DMSO-*d*_6_) *δ* 167.10, 166.68, 164.79, 162.97, 161.05, 149.86, 140.67 (d, *J* = 11.3 Hz), 135.55, 130.85, 130.20 (d, *J* = 10.1 Hz), 129.53, 126.45, 125.89, 124.34, 116.06, 111.22, 110.29 (d, *J* = 21.1 Hz), 107.08 (d, *J* = 26.3 Hz), 40.54, 18.49, 12.40; HRMS calcd for C_16_H_12_ClFN_3_O_2_ [M + H]^+^ 332.0597, found 332.0604.

*3-(5-(chloromethyl)-1,2,4-oxadiazol-3-yl)-N-(4-fluorophenyl)benzamide***12i**. Yellow solid, yield 67.1%, m.p. 132–133 °C; ^1^H-NMR (500 MHz, DMSO-*d*_6_) *δ* 10.57 (s, 1H), 8.60 (s, 1H), 8.23 (d, *J* = 7.9 Hz, 1H), 8.20 (d, *J* = 8.0 Hz, 1H), 7.86–7.80 (m, 2H), 7.76 (t, *J* = 7.8 Hz, 1H), 7.22 (t, *J* = 8.9 Hz, 2H), 5.23 (s, 2H); ^13^C-NMR (126 MHz, DMSO-*d*_6_) *δ* 167.68, 167.21, 165.00, 159.86, 157.95, 150.39, 136.29, 135.74, 131.32, 130.63, 130.06, 126.91, 126.38, 124.90, 122.84 (d, *J* = 7.8 Hz), 115.69 (d, *J* = 22.2 Hz), 111.70, 41.05, 19.00, 12.95; HRMS calcd for C_16_H_12_ClFN_3_O_2_ [M + H]^+^ 332.0597, found 332.0601.

*3-(5-(chloromethyl)-1,2,4-oxadiazol-3-yl)-N-(4-chlorophenyl)benzamide***12j**. Yellow solid, yield 79.3%, m.p. 149–151 °C; ^1^H-NMR (500 MHz, DMSO-*d*_6_) *δ* 8.50 (s, 1H), 8.25 (d, *J* = 7.8 Hz, 1H), 8.14 (s, 1H), 8.07 (d, *J* = 7.9 Hz, 1H), 7.67–7.57 (m, 3H), 7.34 (d, *J* = 8.8 Hz, 2H), 4.78 (s, 2H); ^13^C-NMR (126 MHz, DMSO-*d*_6_) *δ* 167.67, 167.23, 165.08, 150.41, 135.52, 135.35, 131.37, 130.86, 130.20, 130.17, 130.08, 129.09, 128.20, 128.00, 127.05, 126.47, 124.93, 111.70, 41.05, 19.01, 12.96; HRMS calcd for C_16_H_12_Cl_2_N_3_O_2_ [M + H]^+^ 348.0301, found 348.0312.

*3-(5-(chloromethyl)-1,2,4-oxadiazol-3-yl)-N-(4-bromophenyl)benzamide***12k**. Yellow solid, yield 73.4%, m.p. 153–155 °C; ^1^H-NMR (500 MHz, DMSO-*d*_6_) *δ* 10.62 (s, 1H), 8.59 (s, 1H), 8.24 (d, *J* = 7.7 Hz, 1H), 8.20 (d, *J* = 7.8 Hz, 1H), 7.82–7.73 (m, 3H), 7.56 (d, *J* = 8.7 Hz, 2H), 5.23 (s, 2H); ^13^C-NMR (126 MHz, DMSO-*d*_6_) *δ* 167.61, 167.18, 165.27, 150.37, 140.89, 135.99, 133.43, 131.36, 130.77, 130.05, 126.96, 126.40, 124.85, 124.03, 120.34, 119.22, 111.72, 41.04, 18.99, 12.91; HRMS calcd for C_16_H_12_ClBrN_3_O_2_ [M + H]^+^ 391.9796, found 391.9802.

*3-(5-(chloromethyl)-1,2,4-oxadiazol-3-yl)-N-(4-iodophenyl)benzamide***12l**. Yellow solid, yield 68.8%, m.p. 141–143 °C; ^1^H-NMR (500 MHz, DMSO-*d*_6_) *δ* 10.59 (s, 1H), 8.58 (d, *J* = 1.7 Hz, 1H), 8.23 (d, *J* = 7.7 Hz, 1H), 8.19 (d, *J* = 7.8 Hz, 1H), 7.76 (t, *J* = 7.8 Hz, 1H), 7.72 (d, *J* = 8.5 Hz, 2H), 7.65 (d, *J* = 8.4 Hz, 2H), 5.22 (s, 2H); ^13^C-NMR (126 MHz, DMSO-*d*_6_) *δ* 167.69, 167.25, 165.21, 150.41, 138.39, 136.23, 131.42, 130.75, 130.12, 129.04, 128.02, 126.96, 126.41, 124.94, 122.49, 111.70, 41.06, 19.01, 12.98; HRMS calcd for C_16_H_12_ClIN_3_O_2_ [M + H]^+^ 439.9657, found 439.9668.

*3-(5-(chloromethyl)-1,2,4-oxadiazol-3-yl)-N-(2,4-dimethylphenyl)benzamide***12m**. White solid, yield 69.5%, m.p. 114–117 °C; ^1^H-NMR (500 MHz, DMSO-*d*_6_) *δ* 10.11 (s, 1H), 8.63 (s, 1H), 8.22 (d, *J* = 7.8 Hz, 2H), 7.75 (t, *J* = 7.8 Hz, 1H), 7.22 (d, *J* = 7.9 Hz, 1H), 7.10 (s, 1H), 7.04 (d, *J* = 7.9 Hz, 1H), 5.22 (s, 2H), 2.30 (s, 3H), 2.21 (s, 3H); ^13^C-NMR (126 MHz, DMSO-*d*_6_) *δ* 167.68, 167.24, 165.21, 150.41, 138.81, 136.22, 131.96, 131.41, 130.76, 130.12, 126.96, 126.41, 124.93, 122.85, 116.11, 111.70, 41.06, 19.01, 12.97; HRMS calcd for C_18_H_17_ClN_3_O_2_ [M + H]^+^ 342.1004, found 342.1013.

*3-(5-(chloromethyl)-1,2,4-oxadiazol-3-yl)-N-(2,6-dimethylphenyl)benzamide***12n**. White solid, yield 74.4%, m.p. 125–129 °C; ^1^H-NMR (500 MHz, DMSO-*d*_6_) *δ* 10.06 (s, 1H), 8.65 (t, *J* = 1.7 Hz, 1H), 8.28–8.22 (m, 2H), 7.77 (t, *J* = 7.8 Hz, 1H), 7.15 (s, 3H), 5.23 (s, 2H), 2.22 (s, 6H); ^13^C-NMR (126 MHz, DMSO-*d*_6_) *δ* 167.68, 167.24, 165.20, 150.41, 139.29, 137.80, 136.25, 131.41, 130.75, 130.10, 126.96, 126.40, 124.93, 123.09, 111.70, 88.14, 41.06, 19.01, 12.98; HRMS calcd for C_18_H_17_ClN_3_O_2_ [M + H]^+^ 342.1004, found 342.1016.

*3-(5-(chloromethyl)-1,2,4-oxadiazol-3-yl)-N-(3-chloro-2-methylphenyl)benzamide***12o**. White solid, yield 63.7%, m.p. 107–109 °C; ^1^H-NMR (500 MHz, DMSO-*d*_6_) *δ* 10.43 (s, 1H), 8.63 (s, 1H), 8.24 (t, *J* = 8.8 Hz, 1H), 7.77 (t, *J* = 7.8 Hz, 2H), 7.40 (d, *J* = 9.0 Hz, 1H), 7.34 (d, *J* = 7.0 Hz, 1H), 7.28 (t, *J* = 7.9 Hz, 1H), 5.23 (s, 2H), 2.27 (s, 3H); ^13^C-NMR (126 MHz, DMSO-*d*_6_) *δ* 167.72, 167.17, 164.89, 150.38, 136.10, 135.75, 134.05, 131.34, 131.24, 130.47, 130.02, 127.05, 127.02, 126.98, 126.36, 124.89, 111.69, 41.03, 21.02, 19.00, 18.29, 12.93; HRMS calcd for C_17_H_14_Cl_2_N_3_O_2_ [M + H]^+^ 362.0458, found 362.0459.

*3-(5-(chloromethyl)-1,2,4-oxadiazol-3-yl)-N-(3,4-dichlorophenyl)benzamide***12p**. Yellow solid, yield 64.9%, m.p. 138–140 °C; ^1^H-NMR (500 MHz, DMSO-*d*_6_) *δ* 10.72 (s, 1H), 8.59 (s, 1H), 8.23 (d, *J* = 7.8 Hz, 1H), 8.19 (d, *J* = 8.0 Hz, 1H), 8.16 (s, 1H), 7.81–7.74 (m, 2H), 7.61 (d, *J* = 8.8 Hz, 1H), 5.22 (s, 2H); ^13^C-NMR (126 MHz, DMSO-*d*_6_) *δ* 167.70, 167.18, 164.58, 150.38, 136.06, 135.85, 135.54, 131.11, 130.53, 130.11, 128.24, 127.28, 126.88, 126.46, 124.88, 111.69, 41.03, 19.00, 18.53, 12.92; HRMS calcd for C_16_H_11_Cl_3_N_3_O_2_ [M + H]^+^ 381.9911, found 381.9921.

*3-(5-(chloromethyl)-1,2,4-oxadiazol-3-yl)-N-(2,4-difluorophenyl)benzamide***12q**. Yellow solid, yield 63.7%, m.p. 121–124 °C; ^1^H-NMR (500 MHz, DMSO-*d*_6_) *δ* 10.44 (s, 1H), 8.63 (s, 1H), 8.25 (d, *J* = 7.8 Hz, 1H), 8.22 (d, *J* = 7.8 Hz, 1H), 7.77 (t, *J* = 7.8 Hz, 1H), 7.68–7.57 (m, 1H), 7.39 (t, *J* = 9.8 Hz, 1H), 7.15 (t, *J* = 8.5 Hz, 1H), 5.23 (s, 2H); ^13^C-NMR (126 MHz, DMSO-*d*_6_) *δ* 167.67, 167.21, 165.13, 150.39, 138.31, 135.64, 134.30, 132.73, 131.37, 130.77, 130.14, 127.42, 127.39, 127.02, 126.43, 126.40, 124.90, 41.05, 19.00, 15.83, 12.95; HRMS calcd for C_16_H_11_ClF_2_N_3_O_2_ [M + H]^+^ 350.0502, found 350.0511.

#### 3.2.9. Synthesis of Target Compound **13**

5,7-dichloro-4-hydroxyquinoline **5** (0.21 g, 1.00 mmol), intermediate **12** (1.00 mmol), K_2_CO_3_ (0.35 g) and DMF (10 mL) were added to a round bottom flask. The mixture was reacted at 60 °C for 5 h. Afterwards, the mixture was cooled to room temperature and poured into water (100 mL) then extracted with ethyl acetate. The extraction was dried over anhydrous MgSO_4_ and filtered. After that the filtration was concentrated and separated by column chromatography to give target compounds **13**.

*3-(5-(((5,7-dichloroquinolin-4-yl)oxy)methyl)-1,2,4-oxadiazol-3-yl)-N-phenylbenzamide***13a**. Yellow solid, yield 67.6%, HPLC 90.45%, m.p. 221–224 °C; ^1^H-NMR (500 MHz, DMSO-*d*_6_) *δ* 10.46 (s, 1H), 8.48 (s, 1H), 8.17 (d, *J* = 7.5 Hz, 1H), 8.13–8.05 (m, 2H), 7.89 (s, 1H), 7.77 (d, *J* = 7.7 Hz, 2H), 7.73–7.69 (m, 1H), 7.51 (s, 1H), 7.37 (t, *J* = 7.6 Hz, 2H), 7.12 (t, *J* = 7.2 Hz, 1H), 6.20 (d, *J* = 7.8 Hz, 1H), 6.04 (s, 2H); ^13^C-NMR (126 MHz, DMSO-*d*_6_) *δ* 176.00, 175.93, 167.88, 165.02, 144.59, 143.61, 139.33, 136.83, 136.39, 135.00, 131.26, 130.42, 130.03, 129.89, 129.08, 126.78, 126.74, 126.28, 124.38, 121.01, 116.14, 113.04, 48.64; HRMS calcd for C_25_H_17_Cl_2_N_4_O_3_ [M + H]^+^ 491.0672, found 491.0671.

*3-(5-(((5,7-dichloroquinolin-4-yl)oxy)methyl)-1,2,4-oxadiazol-3-yl)-N-(o-tolyl)benzamide***13b**. Yellow solid, yield 64.2%, HPLC 93.24%, m.p. 237–238 °C; ^1^H-NMR (500 MHz, DMSO-*d*_6_) *δ* 10.12 (s, 1H), 8.49 (s, 1H), 8.18 (d, *J* = 7.8 Hz, 1H), 8.11 (d, *J* = 7.9 Hz, 1H), 8.08 (d, *J* = 7.9 Hz, 1H), 7.89 (d, *J* = 1.9 Hz, 1H), 7.71 (t, *J* = 7.8 Hz, 1H), 7.51 (d, *J* = 1.8 Hz, 1H), 7.34 (d, *J* = 7.4 Hz, 1H), 7.28 (d, *J* = 7.4 Hz, 1H), 7.25–7.15 (m, 2H), 6.20 (d, *J* = 7.9 Hz, 1H), 6.03 (s, 2H), 2.22 (s, 3H); ^13^C-NMR (126 MHz, DMSO-*d*_6_) *δ* 175.98, 175.95, 167.88, 164.93, 144.59, 143.60, 136.83, 136.58, 136.01, 134.99, 134.22, 131.17, 130.80, 130.38, 130.09, 127.10, 126.84, 126.74, 126.65, 126.50, 126.30, 121.82, 116.15, 113.03, 48.64, 18.30; HRMS calcd for C_26_H_19_Cl_2_N_4_O_3_ [M + H]^+^ 505.0829, found 505.0831.

*3-(5-(((5,7-dichloroquinolin-4-yl)oxy)methyl)-1,2,4-oxadiazol-3-yl)-N-(m-tolyl)benzamide***13c**. Yellow solid, yield 65.6%, HPLC 93.26%, m.p. 255–258 °C; ^1^H-NMR (500 MHz, DMSO-*d*_6_) *δ* 10.38 (s, 1H), 8.47 (s, 1H), 8.16 (d, *J* = 7.9 Hz, 1H), 8.12–8.06 (m, 2H), 7.89 (d, *J* = 1.8 Hz, 1H), 7.70 (t, *J* = 7.8 Hz, 1H), 7.61 (s, 1H), 7.56 (d, *J* = 8.2 Hz, 1H), 7.51 (d, *J* = 1.8 Hz, 1H), 7.24 (t, *J* = 7.8 Hz, 1H), 6.94 (d, *J* = 7.5 Hz, 1H), 6.20 (d, *J* = 7.9 Hz, 1H), 6.04 (s, 2H), 2.32 (s, 3H); ^13^C-NMR (126 MHz, DMSO-*d*_6_) *δ* 175.99, 175.92, 167.88, 164.93, 144.58, 143.61, 139.25, 138.25, 136.83, 136.43, 134.99, 131.23, 130.39, 130.01, 128.91, 126.76, 126.73, 126.27, 125.07, 121.83, 121.54, 118.18, 116.14, 113.04, 48.64, 21.64; HRMS calcd for C_26_H_19_Cl_2_N_4_O_3_ [M + H]^+^ 505.0829, found 505.0835.

*3-(5-(((5,7-dichloroquinolin-4-yl)oxy)methyl)-1,2,4-oxadiazol-3-yl)-N-(p-tolyl)benzamide***13d**. Yellow solid, yield 63.7%, HPLC 94.00%, m.p. 207–211 °C; ^1^H-NMR (500 MHz, DMSO-*d*_6_) *δ* 10.38 (s, 1H), 8.47 (s, 1H), 8.16 (d, *J* = 7.8 Hz, 1H), 8.12–8.05 (m, 2H), 7.88 (d, *J* = 1.5 Hz, 1H), 7.70 (t, *J* = 7.8 Hz, 1H), 7.64 (d, *J* = 8.3 Hz, 2H), 7.50 (d, *J* = 1.5 Hz, 1H), 7.16 (d, *J* = 8.2 Hz, 2H), 6.20 (d, *J* = 7.9 Hz, 1H), 6.04 (s, 2H), 2.28 (s, 3H); ^13^C-NMR (126 MHz, DMSO-*d*_6_) *δ* 175.97, 175.93, 167.89, 164.79, 144.57, 143.59, 136.83, 136.80, 136.45, 135.00, 133.37, 131.19, 130.32, 129.98, 129.87, 129.45, 126.75, 126.26, 121.83, 121.02, 116.12, 113.04, 48.63, 20.95; HRMS calcd for C_26_H_19_Cl_2_N_4_O_3_ [M + H]^+^ 505.0829, found 505.0832.

*3-(5-(((5,7-dichloroquinolin-4-yl)oxy)methyl)-1,2,4-oxadiazol-3-yl)-N-(4-(tert-butyl)pheny-l)-benzamide***13e**. Yellow solid, yield 65.1%, HPLC 96.69%, m.p. 253–255 °C; ^1^H-NMR (500 MHz, DMSO-*d*_6_) *δ* 10.39 (s, 1H), 8.47 (s, 1H), 8.16 (d, *J* = 6.8 Hz, 1H), 8.09 (t, *J* = 7.2 Hz, 2H), 7.88 (s, 1H), 7.74–7.63 (m, 3H), 7.51 (s, 1H), 7.37 (d, *J* = 7.5 Hz, 2H), 6.20 (d, *J* = 7.3 Hz, 1H), 6.04 (s, 2H), 1.28 (s, 9H); ^13^C-NMR (126 MHz, DMSO-*d*_6_) *δ* 175.99, 175.96, 167.89, 164.84, 146.80, 144.61, 143.60, 136.85, 136.72, 136.42, 135.00, 131.21, 130.37, 130.03, 129.14, 126.75, 126.26, 125.70, 121.82, 120.79, 116.15, 113.04, 48.64, 34.53, 31.65; HRMS calcd for C_29_H_25_Cl_2_N_4_O_3_ [M + H]^+^ 547.1298, found 547.1301.

*3-(5-(((5,7-dichloroquinolin-4-yl)oxy)methyl)-1,2,4-oxadiazol-3-yl)-N-(3-(trifluoromthyl)p-henyl)benzamide***13f**.Yellow solid, yield 57.3%, HPLC 95.23%, m.p. 214–216 °C; ^1^H-NMR (500 MHz, DMSO-*d*_6_) *δ* 10.75 (s, 1H), 8.50 (s, 1H), 8.22 (s, 1H), 8.18 (d, *J* = 7.5 Hz, 1H), 8.12 (d, *J* = 7.5 Hz, 1H), 8.10–8.03 (m, 2H), 7.88 (s, 1H), 7.72 (t, *J* = 7.8 Hz, 1H), 7.61 (t, *J* = 8.1 Hz, 1H), 7.51–7.44 (m, 2H), 6.20 (d, *J* = 7.9 Hz, 1H), 6.04 (s, 2H); ^13^C-NMR (126 MHz, DMSO-*d*_6_) *δ* 176.01, 175.96, 167.83, 165.40, 144.58, 143.58, 140.13, 136.84, 135.84, 135.00, 131.34, 130.75, 130.32, 130.12, 129.85 (q, *J* = 130 Hz), 126.75 (d, *J* = 4.5 Hz), 126.36, 125.65, 124.43, 123.49, 121.82, 120.66, 117.04 (d, *J* = 15 Hz), 116.11, 113.04, 48.64; HRMS calcd for C_26_H_16_Cl_2_F_3_N_4_O_3_ [M + H]^+^ 559.0546, found 559.0549.

*3-(5-(((5,7-dichloroquinolin-4-yl)oxy)methyl)-1,2,4-oxadiazol-3-yl)-N-(2-fluorophenyl)benzamide***13g**. Yellow solid, yield 59.6%, HPLC 96.30%, m.p. 240–242 °C; ^1^H-NMR (500 MHz, DMSO-*d*_6_) *δ* 10.38 (s, 1H), 8.51 (s, 1H), 8.18 (d, *J* = 7.7 Hz, 1H), 8.12 (d, *J* = 7.7 Hz, 1H), 8.09–8.07 (m, 1H), 7.89 (s, 1H), 7.71 (t, *J* = 7.8 Hz, 1H), 7.61 (t, *J* = 7.6 Hz, 1H), 7.50 (s, 1H), 7.34–7.27 (m, 2H), 7.26–7.20 (m, 1H), 6.20 (d, *J* = 7.9 Hz, 1H), 6.04 (s, 2H); ^13^C-NMR (126 MHz, DMSO-*d*_6_) *δ* 176.00, 175.93, 167.85, 165.04, 157.24, 155.28, 144.58, 143.60, 136.83, 135.40, 135.00, 131.35, 130.68, 130.12, 129.88, 127.63, 126.90, 126.73, 126.33, 124.77, 121.83, 116.38, 116.17 (d, *J* = 11.7 Hz), 113.04, 48.64; HRMS calcd for C_25_H_16_Cl_2_FN_4_O_3_ [M + H]^+^ 509.0578, found 509.0581.

*3-(5-(((5,7-dichloroquinolin-4-yl)oxy)methyl)-1,2,4-oxadiazol-3-yl)-N-(3-fluorophenyl)benzamide***13h**. Yellow solid, yield 59.3%, HPLC 91.69%, m.p. 233–236 °C; ^1^H-NMR (500 MHz, DMSO-*d*_6_) *δ* 10.63 (s, 1H), 8.47 (s, 1H), 8.16 (d, *J* = 7.6 Hz, 1H), 8.12 (d, *J* = 7.6 Hz, 1H), 8.08 (d, *J* = 5.8 Hz, 1H), 7.88 (s, 1H), 7.77–7.68 (m, 2H), 7.55 (d, *J* = 7.8 Hz, 1H), 7.50 (s, 1H), 7.43–7.36 (m, 1H), 6.95 (t, *J* = 7.0 Hz, 1H), 6.20 (d, *J* = 7.5 Hz, 1H), 6.04 (s, 2H); ^13^C-NMR (126 MHz, DMSO-*d*_6_) *δ* 175.99, 175.96, 167.83, 165.29, 163.45, 161.53, 144.58, 143.58, 141.09 (d, *J* = 10.9 Hz), 136.84, 136.03, 135.01, 134.18, 131.30, 130.69 (d, *J* = 11.3 Hz), 130.09, 129.86, 126.76 (d, *J* = 7.1 Hz), 121.82, 116.61, 116.11, 113.04, 110.82 (d, *J* = 21.0 Hz), 107.64 (d, *J* = 26.0 Hz), 48.63; HRMS calcd for C_25_H_16_Cl_2_FN_4_O_3_ [M + H]^+^ 509.0578, found 509.0583.

*3-(5-(((5,7-dichloroquinolin-4-yl)oxy)methyl)-1,2,4-oxadiazol-3-yl)-N-(4-fluorophenyl)benzamide***13i**. Yellow solid, yield 61.6%, HPLC 92.59%, m.p. 246–248 °C; ^1^H-NMR (500 MHz, DMSO-*d*_6_) *δ* 10.51 (s, 1H), 8.47 (s, 1H), 8.16 (d, *J* = 7.9 Hz, 1H), 8.12–8.06 (m, 2H), 7.89–7.86 (s, 1H), 7.80–7.75 (m, 2H), 7.71 (t, *J* = 7.7 Hz, 1H), 7.50 (s, 1H), 7.20 (t, *J* = 8.6 Hz, 2H), 6.20 (d, *J* = 7.9 Hz, 1H), 6.04 (s, 2H); ^13^C-NMR (126 MHz, DMSO-*d*_6_) *δ* 175.98, 175.96, 167.86, 165.49, 164.94, 159.86, 157.95, 144.58, 143.59, 136.84, 136.19, 135.65 (d, *J* = 2.3 Hz), 135.01, 134.19, 131.21, 130.47, 129.95 (d, *J* = 22.4 Hz), 126.74, 126.30, 122.88 (d, *J* = 7.9 Hz), 121.82, 116.11, 115.65 (d, *J* = 22.2 Hz), 113.04, 48.63; HRMS calcd for C_25_H_16_Cl_2_FN_4_O_3_ [M + H]^+^ 509.0578, found 509.0576.

*3-(5-(((5,7-dichloroquinolin-4-yl)oxy)methyl)-1,2,4-oxadiazol-3-yl)-N-(4-chlorophenyl)benzamide***13j**. Yellow solid, yield 61.7%, HPLC 92.14%, m.p. 254–257 °C; ^1^H-NMR (500 MHz, DMSO-*d*_6_) *δ* 10.58 (s, 1H), 8.47 (s, 1H), 8.16 (d, *J* = 7.9 Hz, 1H), 8.11 (d, *J* = 7.9 Hz, 1H), 8.09–8.06 (m, 1H), 7.88 (d, *J* = 1.8 Hz, 1H), 7.80 (d, *J* = 8.9 Hz, 2H), 7.71 (t, *J* = 7.8 Hz, 1H), 7.51 (d, *J* = 1.8 Hz, 1H), 7.42 (d, *J* = 8.9 Hz, 2H), 6.20 (d, *J* = 7.9 Hz, 1H), 6.04 (s, 2H); ^13^C-NMR (126 MHz, DMSO-*d*_6_) *δ* 175.99, 175.95, 167.84, 165.11, 144.58, 143.59, 138.29, 136.84, 136.10, 135.00, 131.28, 130.57, 130.07, 128.99, 128.04, 126.78, 126.74, 126.31, 122.51, 121.82, 116.12, 113.04, 48.63; HRMS calcd for C_25_H_16_Cl_3_N_4_O_3_ [M + H]^+^ 525.0282, found 525.0283.

*3-(5-(((5,7-dichloroquinolin-4-yl)oxy)methyl)-1,2,4-oxadiazol-3-yl)-N-(4-bromophenyl)benzamide***13k**. Yellow solid, yield 63.8%, HPLC 91.72%, m.p. 263–264 °C; ^1^H-NMR (500 MHz, DMSO-*d*_6_) *δ* 10.57 (s, 1H), 8.47 (s, 1H), 8.15 (d, *J* = 7.5 Hz, 1H), 8.11 (d, *J* = 7.7 Hz, 1H), 8.08 (d, *J* = 7.5 Hz, 1H), 7.87 (s, 1H), 7.75 (d, *J* = 8.5 Hz, 2H), 7.73–7.68 (m, 1H), 7.54 (d, *J* = 8.5 Hz, 2H), 7.49 (s, 1H), 6.20 (d, *J* = 7.8 Hz, 1H), 6.04 (s, 2H); ^13^C-NMR (126 MHz, DMSO-*d*_6_) *δ* 175.99, 175.96, 167.84, 165.11, 144.58, 143.59, 138.72, 136.84, 136.09, 135.01, 131.90, 131.28, 130.57, 130.06, 129.87, 126.78, 126.74, 126.31, 122.87, 121.83, 116.12, 113.04, 48.63; HRMS calcd for C_25_H_16_Cl_2_BrN_4_O_3_ [M + H]^+^ 568.9777, found 568.9778.

*3-(5-(((5,7-dichloroquinolin-4-yl)oxy)methyl)-1,2,4-oxadiazol-3-yl)-N-(4-iodophenyl)benzamide***13l**. Yellow solid, yield 64.8%, HPLC 90.03%, m.p. 257–259 °C; ^1^H-NMR (500 MHz, DMSO-*d*_6_) *δ* 10.54 (s, 1H), 8.46 (s, 1H), 8.15 (d, *J* = 7.8 Hz, 1H), 8.11 (d, *J* = 7.8 Hz, 1H), 8.07 (d, *J* = 8.1 Hz, 1H), 7.88 (s, 1H), 7.74–7.68 (m, 3H), 7.61 (d, *J* = 8.4 Hz, 2H), 7.51 (s, 1H), 6.20 (d, *J* = 7.8 Hz, 1H), 6.03 (s, 2H); ^13^C-NMR (126 MHz, DMSO-*d*_6_) *δ* 175.98, 167.83, 165.11, 144.60, 143.58, 139.20, 137.76, 136.86, 136.11, 135.00, 131.28, 130.57, 130.08, 126.76, 126.30, 123.13, 121.81, 116.12, 113.03, 88.10, 48.64; HRMS calcd for C_25_H_16_Cl_2_IN_4_O_3_ [M + H]^+^ 616.9639, found 616.9642.

*3-(5-(((5,7-dichloroquinolin-4-yl)oxy)methyl)-1,2,4-oxadiazol-3-yl)-N-(2,4-dimethylphenyl)benzamide***13m**. Yellow solid, yield 66.6%, HPLC 94.60%, m.p. 211–214 °C; ^1^H-NMR (500 MHz, DMSO-*d*_6_) *δ* 10.04 (s, 1H), 8.49 (s, 1H), 8.18 (d, *J* = 7.2 Hz, 1H), 8.12–8.05 (m, 2H), 7.89 (s, 1H), 7.69 (t, *J* = 7.7 Hz, 1H), 7.50 (s, 1H), 7.20 (d, *J* = 7.7 Hz, 1H), 7.08 (s, 1H), 7.02 (d, *J* = 7.5 Hz, 1H), 6.20 (d, *J* = 7.8 Hz, 1H), 6.03 (s, 2H), 2.28 (s, 3H), 2.18 (s, 3H); ^13^C-NMR (126 MHz, DMSO-*d*_6_) *δ* 175.97, 175.94, 167.90, 164.93, 144.58, 143.61, 136.83, 136.08, 135.75, 135.00, 134.02, 133.98, 131.32, 131.13, 130.30, 130.05, 127.01, 126.83, 126.73, 126.28, 121.83, 116.15, 113.04, 48.64, 20.99, 18.23; HRMS calcd for C_27_H_21_Cl_2_N_4_O_3_ [M + H]^+^ 519.0985, found 519.0986.

*3-(5-(((5,7-dichloroquinolin-4-yl)oxy)methyl)-1,2,4-oxadiazol-3-yl)-N-(2,6-dimethylphenyl)benzamide***13n**. Yellow solid, yield 63.5%, HPLC 95.45%, m.p. 227–228 °C; ^1^H-NMR (500 MHz, DMSO-*d*_6_) *δ* 10.00 (s, 1H), 8.51 (s, 1H), 8.21 (d, *J* = 7.9 Hz, 1H), 8.11 (d, *J* = 7.9 Hz, 1H), 8.09–8.06 (m, 1H), 7.88 (d, *J* = 1.8 Hz, 1H), 7.71 (t, *J* = 7.8 Hz, 1H), 7.50 (d, *J* = 1.8 Hz, 1H), 7.13 (s, 3H), 6.20 (d, *J* = 7.9 Hz, 1H), 6.04 (s, 2H), 2.18 (s, 6H); ^13^C-NMR (126 MHz, DMSO-*d*_6_) *δ* 175.95, 167.89, 164.65, 144.57, 143.59, 136.83, 136.00, 135.87, 135.46, 135.00, 130.98, 130.34, 130.13, 129.88, 128.21, 127.25, 126.76, 126.72, 126.38, 121.82, 116.14, 113.03, 48.63, 18.47; HRMS calcd for C_27_H_21_Cl_2_N_4_O_3_ [M + H]^+^ 519.0985, found 519.0983.

*3-(5-(((5,7-dichloroquinolin-4-yl)oxy)methyl)-1,2,4-oxadiazol-3-yl)-N-(3-chloro-2-methylph-enyl)benzamide***13o**. Yellow solid, yield 49.8%, HPLC 93.20%, m.p. 241–244 °C; ^1^H-NMR (500 MHz, DMSO-*d*_6_) *δ* 10.37 (s, 1H), 8.49 (s, 1H), 8.18 (d, *J* = 7.8 Hz, 1H), 8.12 (d, *J* = 7.8 Hz, 1H), 8.07 (d, *J* = 7.9 Hz, 1H), 7.88 (d, *J* = 1.9 Hz, 1H), 7.72 (t, *J* = 7.8 Hz, 1H), 7.51 (d, *J* = 1.8 Hz, 1H), 7.38 (d, *J* = 7.7 Hz, 1H), 7.32 (d, *J* = 7.5 Hz, 1H), 7.26 (t, *J* = 7.9 Hz, 1H), 6.20 (d, *J* = 7.9 Hz, 1H), 6.03 (s, 2H), 2.23 (s, 3H); ^13^C-NMR (126 MHz, DMSO-*d*_6_) *δ* 176.04, 175.93, 167.84, 165.13, 144.60, 143.63, 138.26, 136.83, 135.62, 134.99, 134.28, 132.71, 131.27, 130.59, 130.16, 127.38, 126.86, 126.74, 126.38, 121.83, 116.17, 113.04, 99.99, 48.64, 15.77; HRMS calcd for C_26_H_18_Cl_3_N_4_O_3_ [M + H]^+^ 539.0439, found 539.0446.

*3-(5-(((5,7-dichloroquinolin-4-yl)oxy)methyl)-1,2,4-oxadiazol-3-yl)-N-(3,4-dichlorophenyl)benzamide* **13p**. Yellow solid, yield 51.4%, HPLC 94.83%, m.p. 265–269 °C; ^1^H-NMR (500 MHz, DMSO-*d*_6_) *δ* 10.70 (s, 1H), 8.47 (s, 1H), 8.17–8.10 (m, 3H), 8.08 (d, *J* = 7.9 Hz, 1H), 7.88 (d, *J* = 2.0 Hz, 1H), 7.77–7.69 (m, 2H), 7.62 (d, *J* = 8.8 Hz, 1H), 7.51 (d, *J* = 2.0 Hz, 1H), 6.20 (d, *J* = 7.8 Hz, 1H), 6.04 (s, 2H); ^13^C-NMR (126 MHz, DMSO-*d*_6_) *δ* 176.02, 175.96, 167.79, 165.30, 144.59, 143.58, 139.46, 136.84, 135.72, 135.00, 131.34, 130.99, 130.79, 130.14, 126.77, 126.74, 126.36, 125.87, 122.09, 121.82, 120.85, 116.13, 113.04, 48.64; HRMS calcd for C_25_H_15_Cl_4_N_4_O_3_ [M + H]^+^ 558.9893, found 558.9899.

*3-(5-(((5,7-dichloroquinolin-4-yl)oxy)methyl)-1,2,4-oxadiazol-3-yl)-N-(2,4-difluorophenyl)benzamide* **13q**. Brown solid, yield 47.9%, HPLC 93.53%, m.p. 235–237 °C; ^1^H-NMR (500 MHz, DMSO-*d*_6_) *δ* 10.38 (s, 1H), 8.50 (s, 1H), 8.17 (d, *J* = 7.8 Hz, 1H), 8.12 (d, *J* = 7.9 Hz, 1H), 8.07 (d, *J* = 7.9 Hz, 1H), 7.88 (d, *J* = 1.8 Hz, 1H), 7.71 (t, *J* = 7.8 Hz, 1H), 7.63–7.56 (m, 1H), 7.51 (d, *J* = 1.8 Hz, 1H), 7.40–7.33 (m, 1H), 7.13 (t, *J* = 8.0 Hz, 1H), 6.20 (d, *J* = 7.9 Hz, 1H), 6.03 (s, 2H); ^13^C-NMR (126 MHz, DMSO-*d*_6_) *δ* 176.00, 175.97, 167.82, 165.14, 159.30 (d, *J* = 11.5 Hz), 157.55 (d, *J* = 12.8 Hz), 155.56 (d, *J* = 12.7 Hz), 144.60, 143.58, 136.85, 135.17, 134.99, 131.33, 130.75, 130.17, 129.00 (d, *J* = 12.2 Hz), 126.84, 126.74, 126.35, 121.81, 116.12, 113.03, 111.70 (d, *J* = 18.6 Hz), 104.85, 48.65; HRMS calcd for C_25_H_15_Cl_2_F_2_N_4_O_3_ [M + H]^+^ 527.0484, found 527.0487.

### 3.3. Biological Activity and Toxicity Determination

The fungicidal activities were investigated in the National Pesticide Engineering Research Center, Nankai University, according to reference [25], and the results of the activity test are shown in Table 2. The toxicity was determined according to Ref. [26].

Through acute exposure, we assessed the toxicity of compound **13p** on zebrafish embryo. According to the preliminary exposure experiments, a series of gradient concentrations of compound **13p** was set on the basis of mortality rates in the range of 10–95%. LC_50_ values for zebrafish embryos exposed to compound **13p** from 24 to 96 hpf: control (0 mg/L of **13p**), 5, 10, 20 mg/L of **13p**. The LC_50_ (median lethal concentration) values were computed by the Boltzmann equation [26,27]. The observational indexes included mortality rate and teratogenic effects.

## 4. Conclusions

In conclusion, a total of 17 novel benzamides containing quinoline-linked 1,2,4-oxadiazole moiety were designed using splicing principle of active substructures and synthesized via Williamson ether synthesis. The structures of target compounds were confirmed by ^1^H NMR, ^13^C NMR, and HRMS. The bioassay results showed that **13a**–**13q** displayed certain inhibitory activity against 10 fungi tested, especially **13f** and **13p**. It is worth mentioning that the fungicidal activities of **13f** and **13p** to *Sclerotinia sclerotiorum* were better than quinoxyfen (14.19 mg/L) with EC_50_ of 6.67 mg/L and 5.17 mg/L, and their inhibition rates were equal (77.8%) or higher (86.1%) than quinoxyfen (77.8%) at 50 mg/L. Moreover, the acute toxicity of **13p** was 19.42 mg/L, which was classified as a low-toxic compound. Hence, these compounds could potentially be lead compounds for further study.

## Figures and Tables

**Table 1:** Effects of reaction conditions on the synthesis of compound **5**.

Entry	Oxidant	Solvent	Reaction Time/h	Yield/%
1	H_2_O_2_	MeCN	4	/
2	MnO_2_	THF	10	43.5
3	K_2_S_2_O_8_/H_2_SO_4_	MeCN	4	86.5

**Table 2:** Fungicidal activities of compounds **13a**–**13q** at 50 mg/L.

Compounds	R	Fungicidal Activities (Inhibition Rate %)									
		*AS*	*GZ*	*PO*	*PC*	*SS*	*BC*	*RS*	*FO*	*CA*	*PP*
**13a**	H	7.1	3.2	20.0	25.0	75.0	30.4	6.3	11.5	6.7	15.4
**13b**	2-CH_3_	14.3	16.1	6.7	18.8	72.2	21.7	6.3	11.5	13.3	26.9
**13c**	3-CH_3_	21.4	9.7	20.0	9.4	69.4	30.4	6.3	11.5	6.7	11.5
**13d**	4-CH_3_	21.4	9.7	20.0	9.4	75.0	34.8	25.0	15.4	6.7	11.5
**13e**	4-t-Bu	35.7	19.4	33.3	18.8	55.6	17.4	25.0	15.4	13.3	30.8
**13f**	3-CF_3_	21.4	32.3	20.0	18.8	77.8	21.7	6.3	3.8	20.0	15.4
**13g**	2-F	21.4	45.2	20.0	18.8	69.4	21.7	8.3	7.7	6.7	34.6
**13h**	3-F	14.3	38.7	20.0	31.3	69.4	34.8	4.2	7.7	6.7	38.5
**13i**	4-F	21.4	6.5	20.0	34.4	47.2	8.7	10.4	3.8	13.3	38.5
**13j**	4-Cl	21.4	3.2	6.7	31.3	55.6	30.4	4.2	3.8	6.7	11.5
**13k**	4-Br	35.7	19.4	33.3	18.8	55.6	21.7	39.6	19.2	20.0	26.9
**13l**	4-I	35.7	29.0	33.3	31.3	55.6	8.7	35.4	3.8	13.3	19.2
**13m**	2,4-di-CH_3_	35.7	45.2	20.0	28.1	50.0	17.4	4.2	7.7	20.0	19.2
**13n**	2,6-di-CH_3_	35.7	25.8	20.0	18.8	58.3	34.8	18.8	7.7	13.3	38.5
**13o**	3-Cl-2-CH_3_	35.7	38.7	33.3	18.8	75.0	30.4	8.3	7.7	20.0	38.5
**13p**	3-Cl-4-Cl	7.1	48.4	33.3	31.3	86.1	21.7	35.4	11.5	26.7	38.5
**13q**	2-F-4-F	14.3	32.3	20.0	25.0	66.7	13.0	10.4	7.7	13.3	19.2
quinoxyfen		35.7	45.2	46.7	9.4	77.8	30.4	25.0	42.3	33.3	38.5

Note: *Alternaria solani* (*AS*), *Gibberella zeae* (*GZ*), *Pyricularia oryae* (*PO*), *Phytophthora capsica* (*PC*), *Sclerotinia sclerotiorum* (*SS*), *Botrytis cinerea* (*BC*), *Riziocotinia solani* (*RS*), *Fusarium oxysporum* (*FO*), *Cercospora arachidicola* (*CA*), *Physalospora piricola* (*PP*). All the data were determined three times.

**Table 3:** EC_50_ of compounds **13f**, **13p** and quinoxyfen to *Sclerotinia sclerotiorum* (*SS*).

Compounds	y = a + bx	r^2^	EC_50_/(mg·L^−1^)
**13f**	y = 1.0563x + 4.1298	0.9845	6.67
**13p**	y = 1.0992x + 4.2153	0.9938	5.17
quinoxyfen	y = 1.5356x + 3.2309	0.9784	14.19

## Supplementary Materials

The following supporting information can be downloaded at: https://www.mdpi.com/article/10.3390/molecules27123946/s1. Figures S1–S17: ^1^H-NMR spectra of **13a**–**q**; Figures S18–S34: ^13^C-NMR spectra of **13a**–**q**; Figures S35–S51: ESI-HRMS spectra of **13a**–**q**.

## Sample Availability

Samples of the compounds are not available from the authors.
