# Supplementary material for: Benzamides Substituted with Quinoline-Linked 1,2,4-Oxadiazole: Synthesis, Biological Activity and Toxicity to Zebrafish Embryo
Source: Molecules. 2022 Jun 20;27(12):3946. doi: 10.3390/molecules27123946 (PMC9229796; doi:10.3390/molecules27123946)
Supplement: Supplementary file 1 [file molecules-27-03946-s001.zip › molecules-1760549-supplementary.pdf]

Supporting Information

Benzamides Substituted with Quinoline-Linked 1,2,4-Oxadiazole: Synthesis, Biological Activity and Toxicity to Zebrafish Embryo

Bin-Long Sun, Ying-Ying Wang, Sen Yang, Min-Ting Tu, Ying-Ying Shao, Yi Hua, Yi Zhou and Cheng-Xia Tan *

1. ^{1}H NMR spectra of **13a~13q**……………………………………S1-S17
2. ^{13}C NMR spectra of **13a~13q**……………………………………S18-S34
3. ESI-HRMS spectra of **13a~13q**……………………………………S35-S51

1. ^{1}H NMR spectra of **13a~13q**

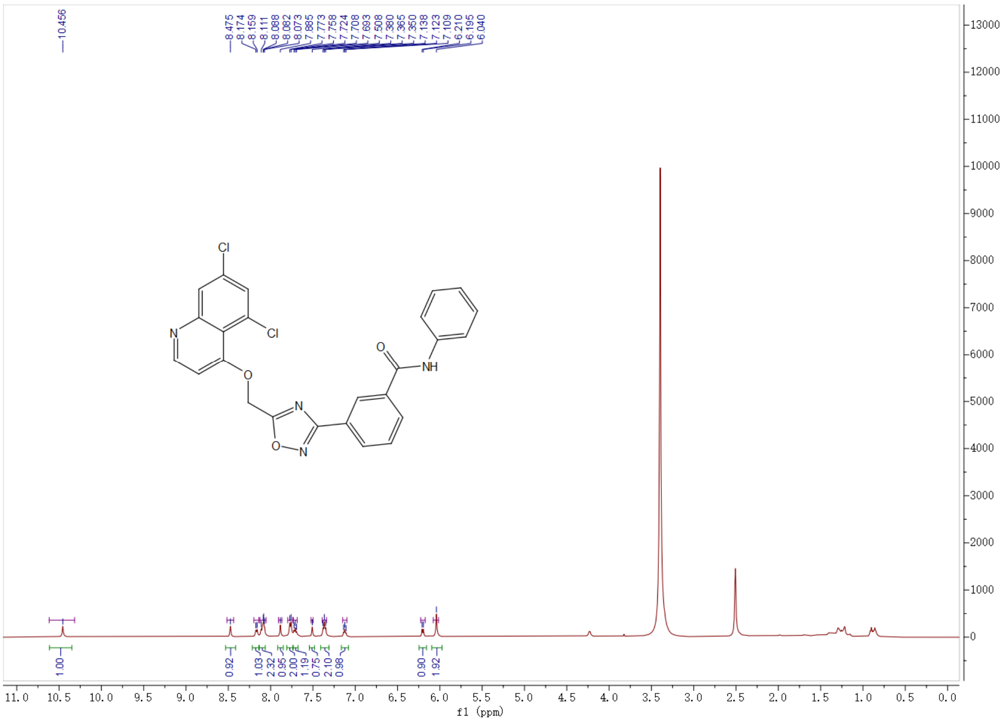

Figure S1. ^{1}H NMR spectra of **13a.**

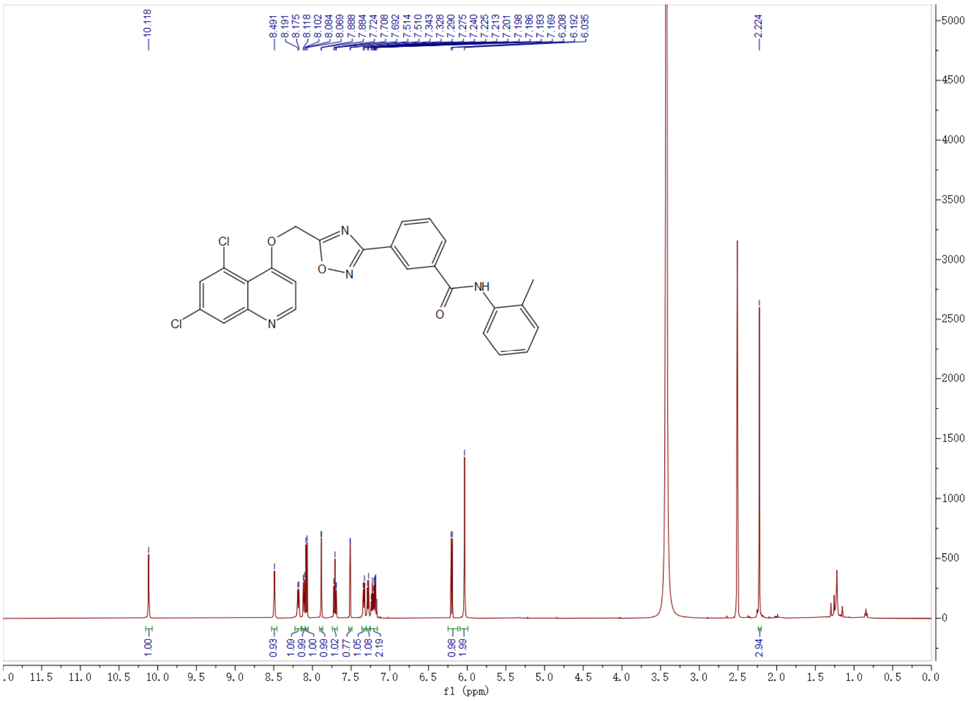

Figure S2. ^{1}H NMR spectra of **13b.**

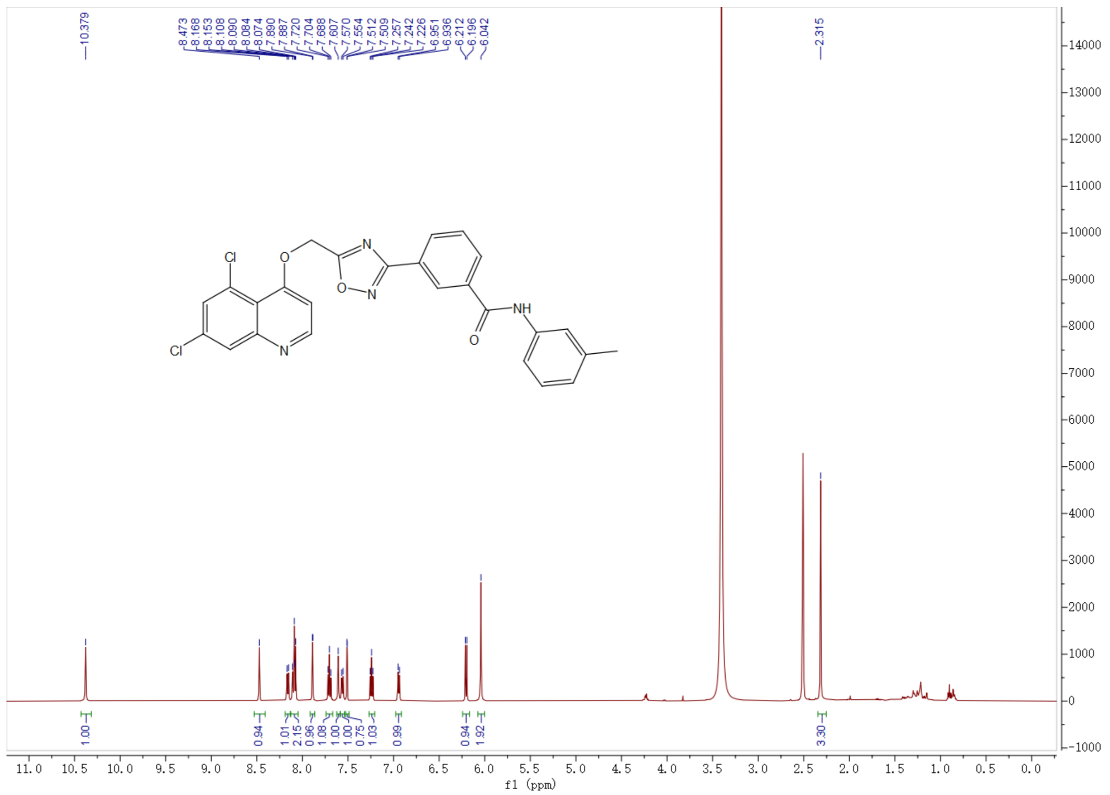

Figure S3. ^{1}H NMR spectra of **13c.**

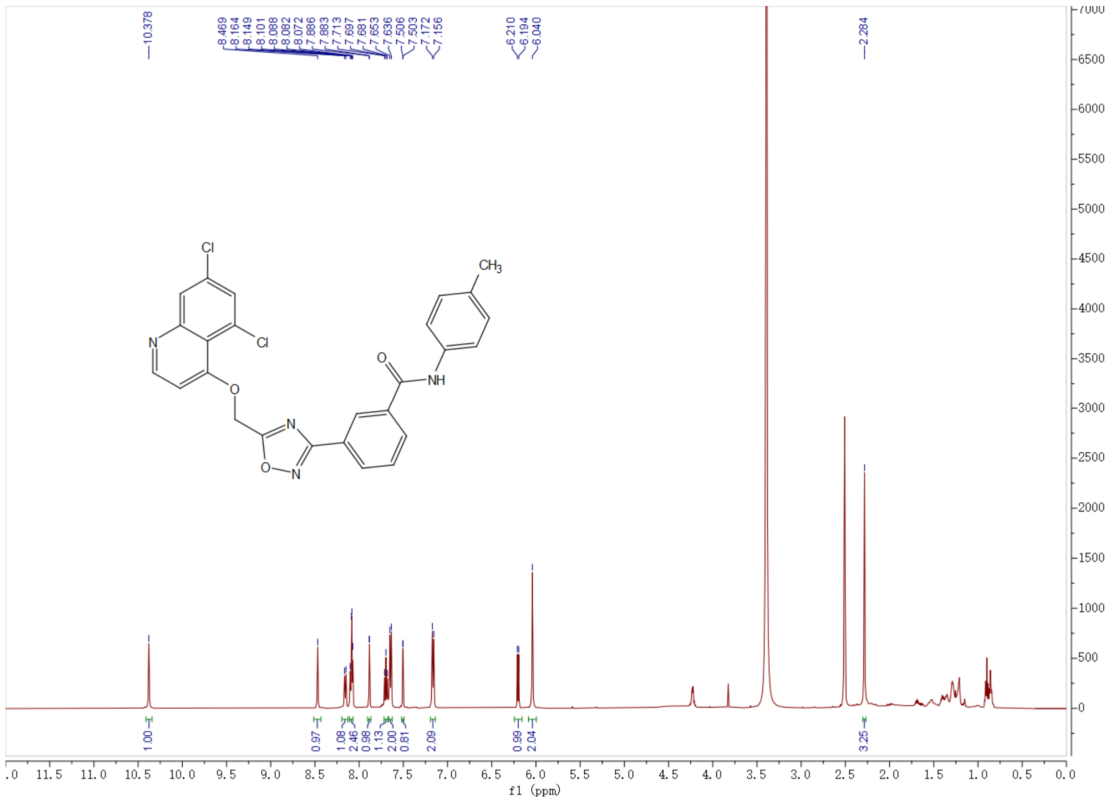

Figure S4. ^{1}H NMR spectra of **13d.**

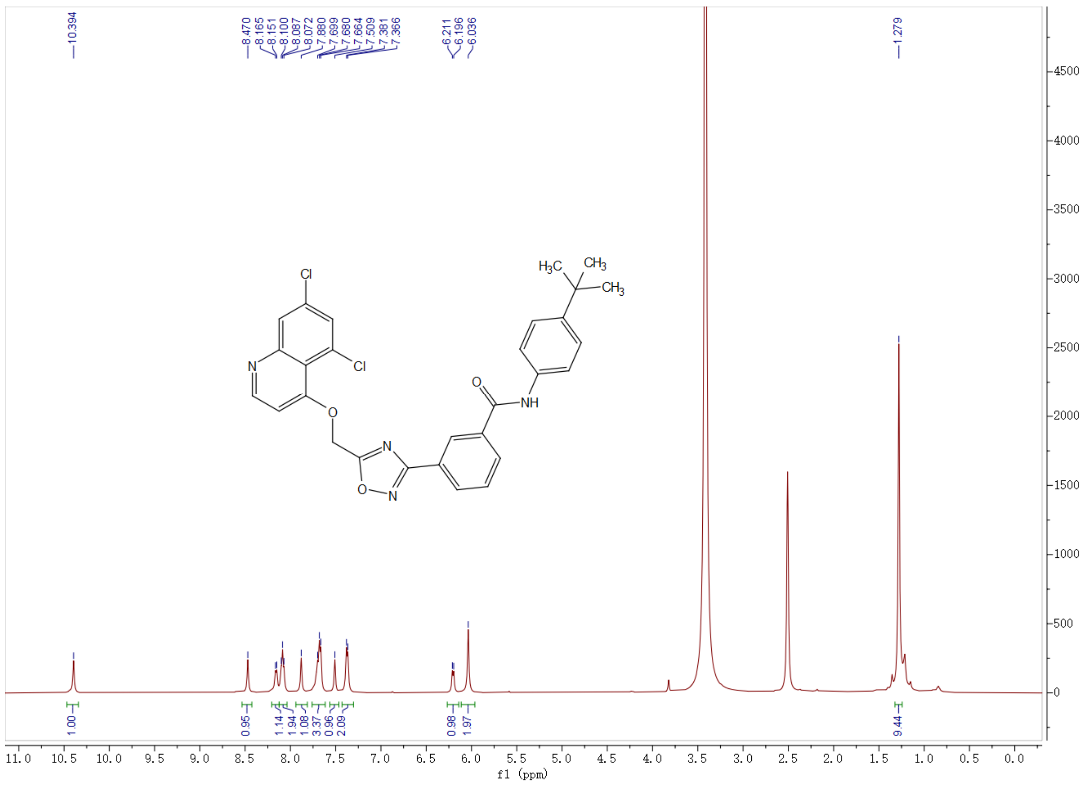

Figure S5. ^{1}H NMR spectra of **13e.**

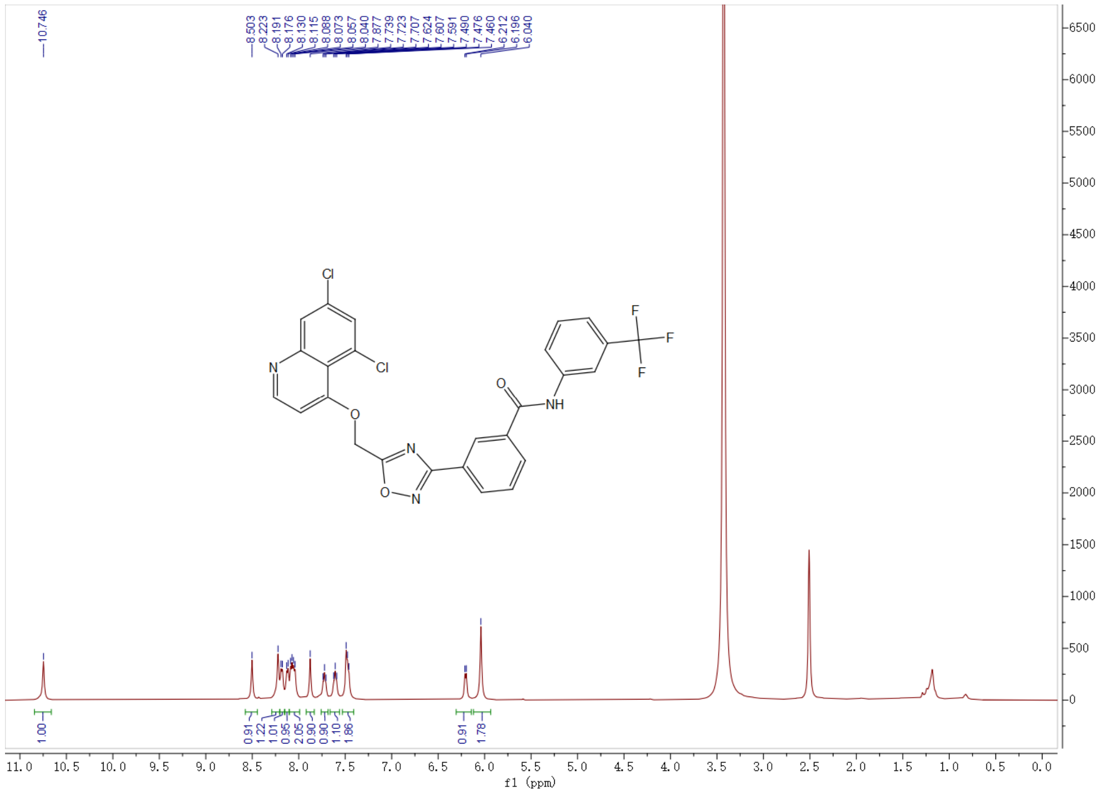

Figure S6. ^{1}H NMR spectra of **13f.**

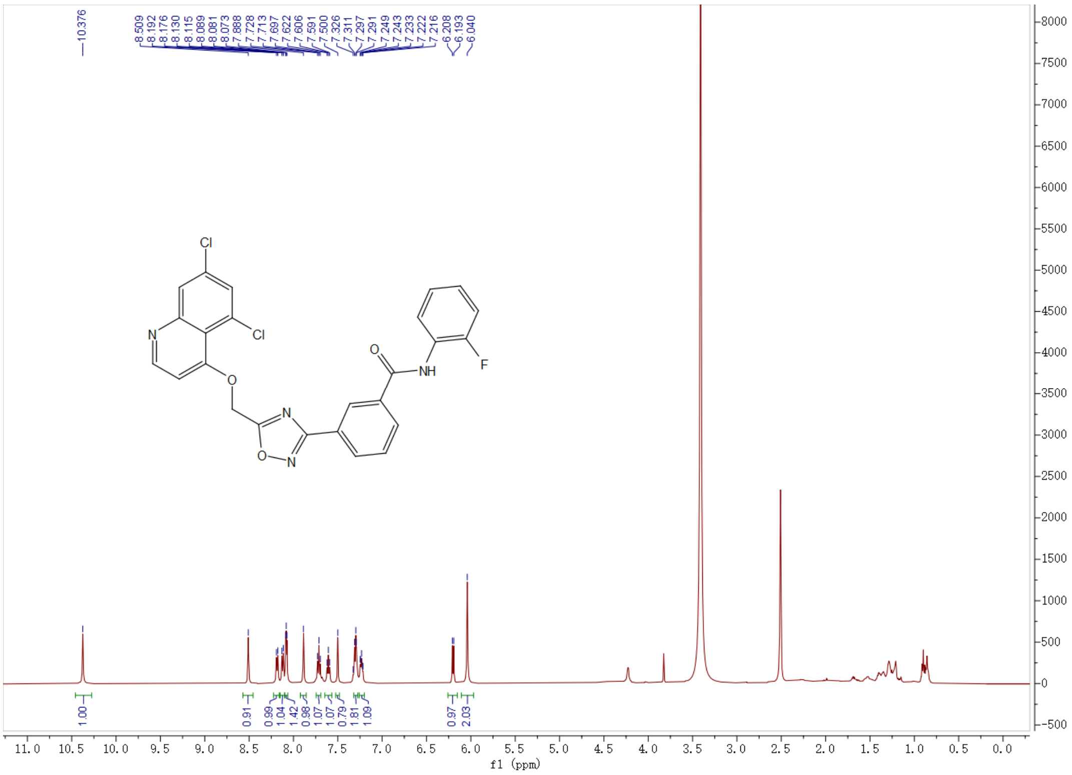

Figure S7. ¹H NMR spectra of **13g.**

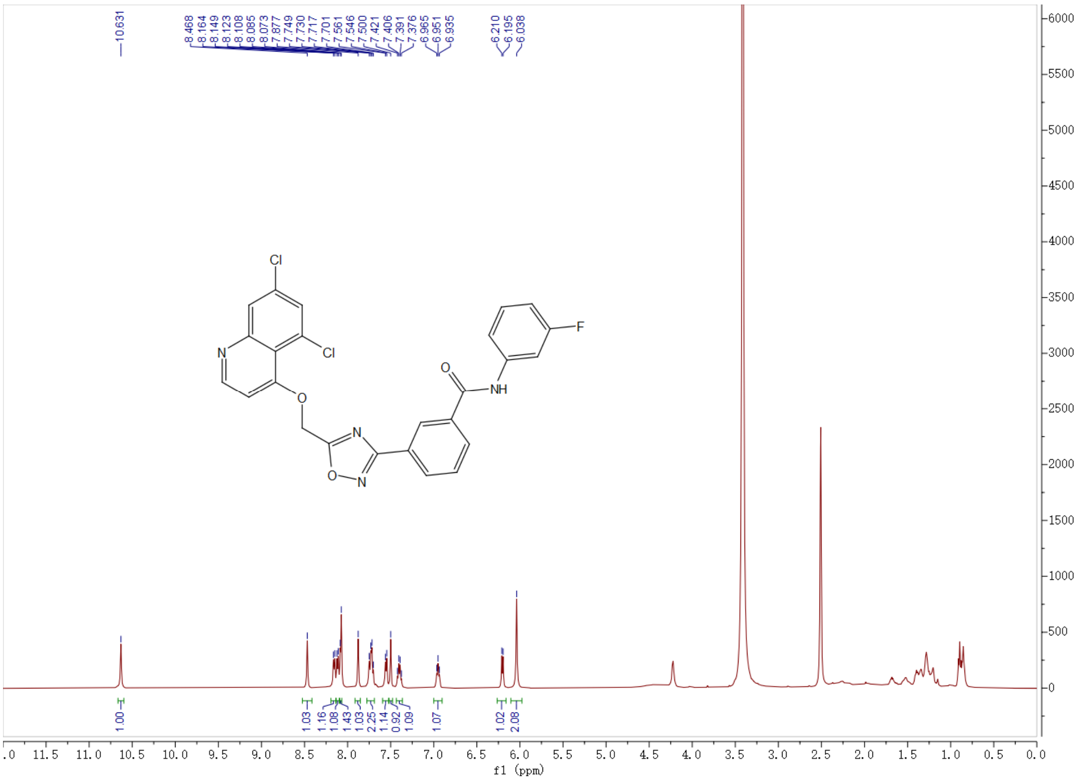

Figure S8. ¹H NMR spectra of **13h.**

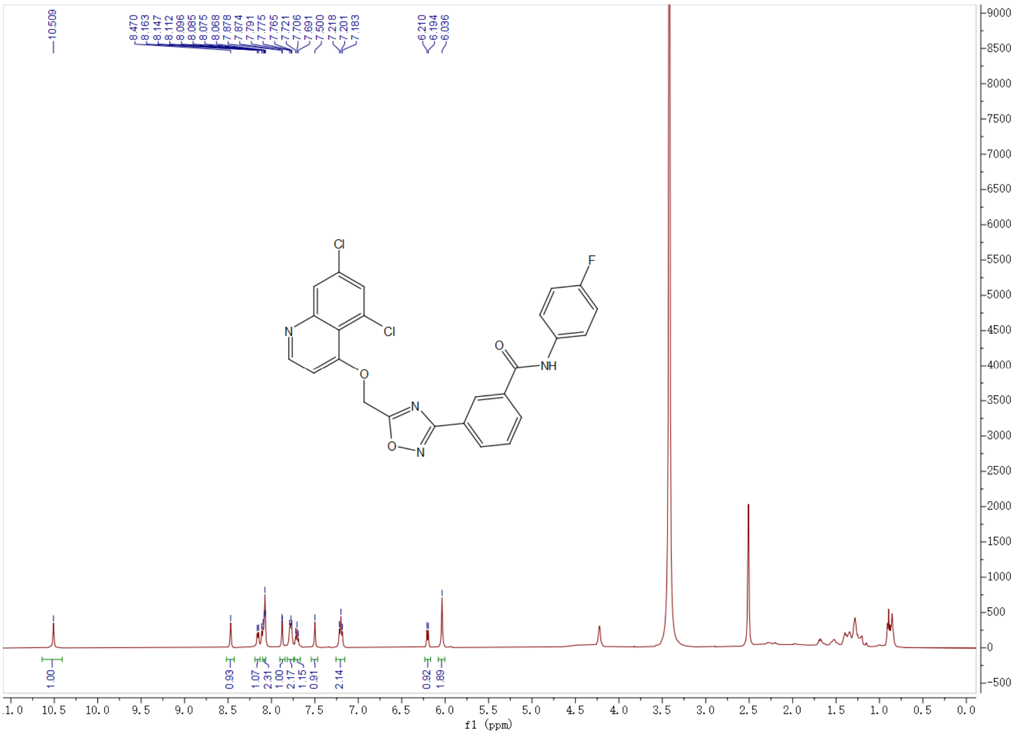

Figure S9. ^{1}H NMR spectra of **13i.**

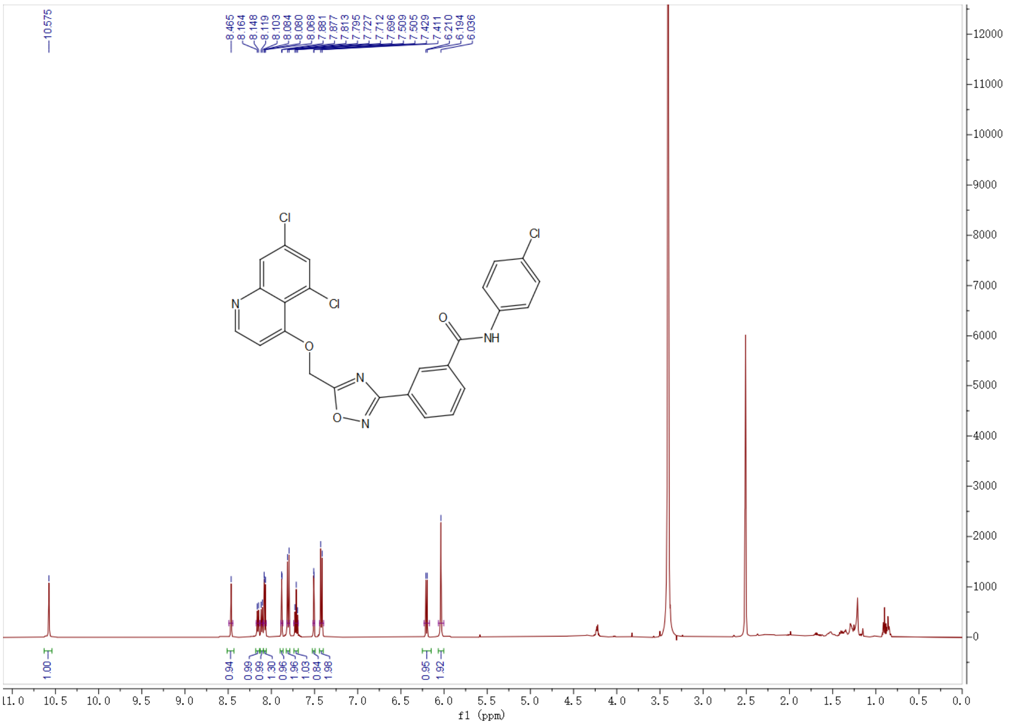

Figure S10. ^{1}H NMR spectra of **13j.**

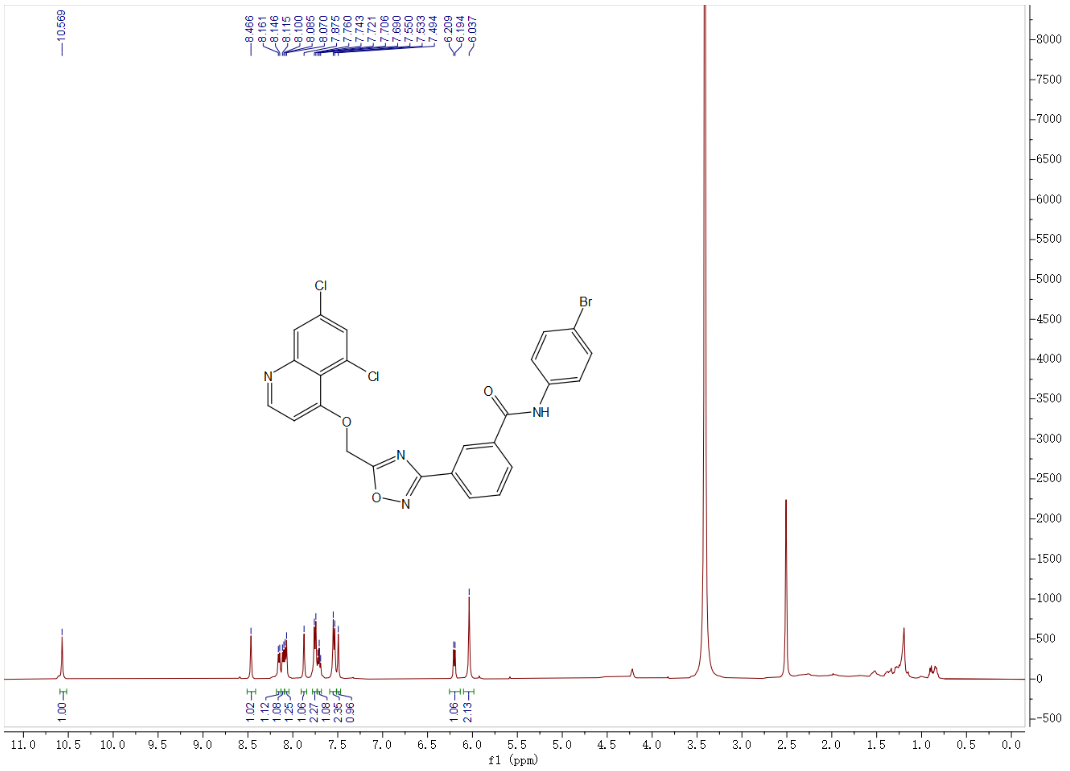

Figure S11. ^{1}H NMR spectra of 13**k.**

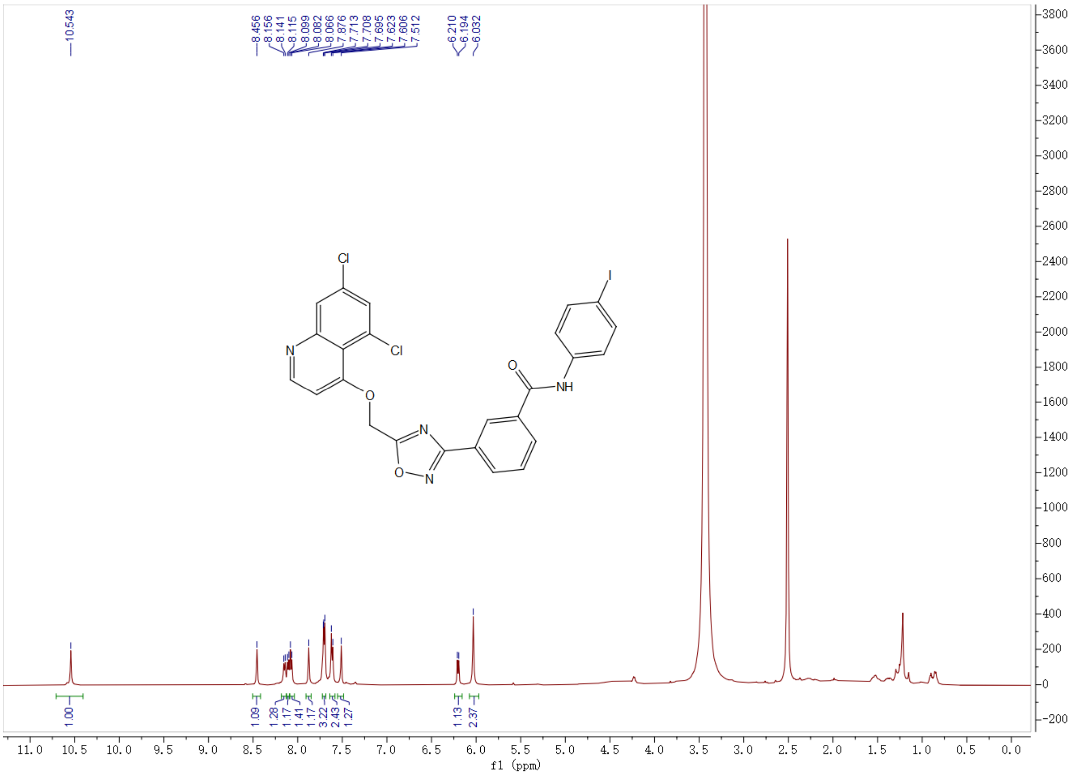

Figure S12. ^{1}H NMR spectra of **13l.**

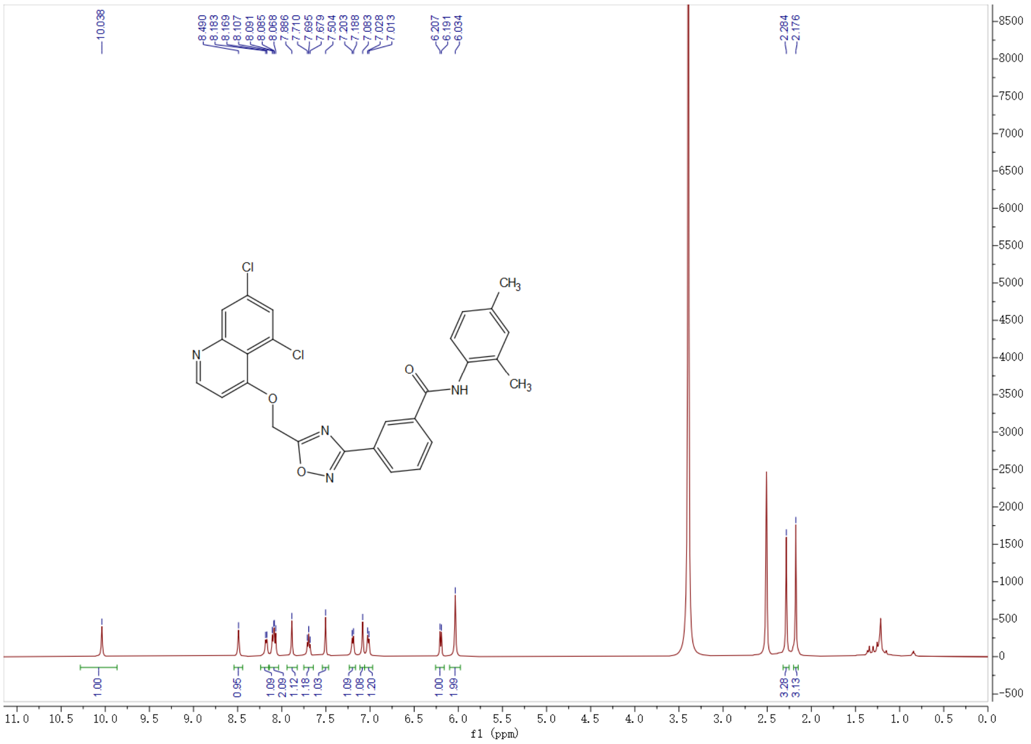

Figure S13. ^{1}H NMR spectra of **13m.**

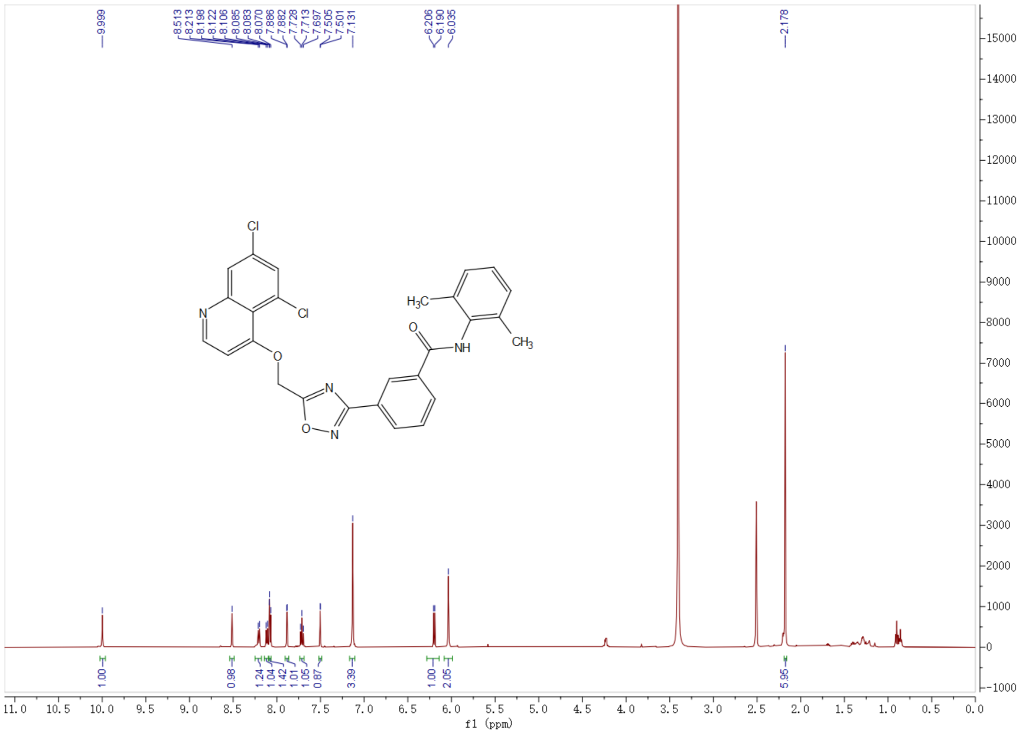

Figure S14. ^{1}H NMR spectra of **13n.**

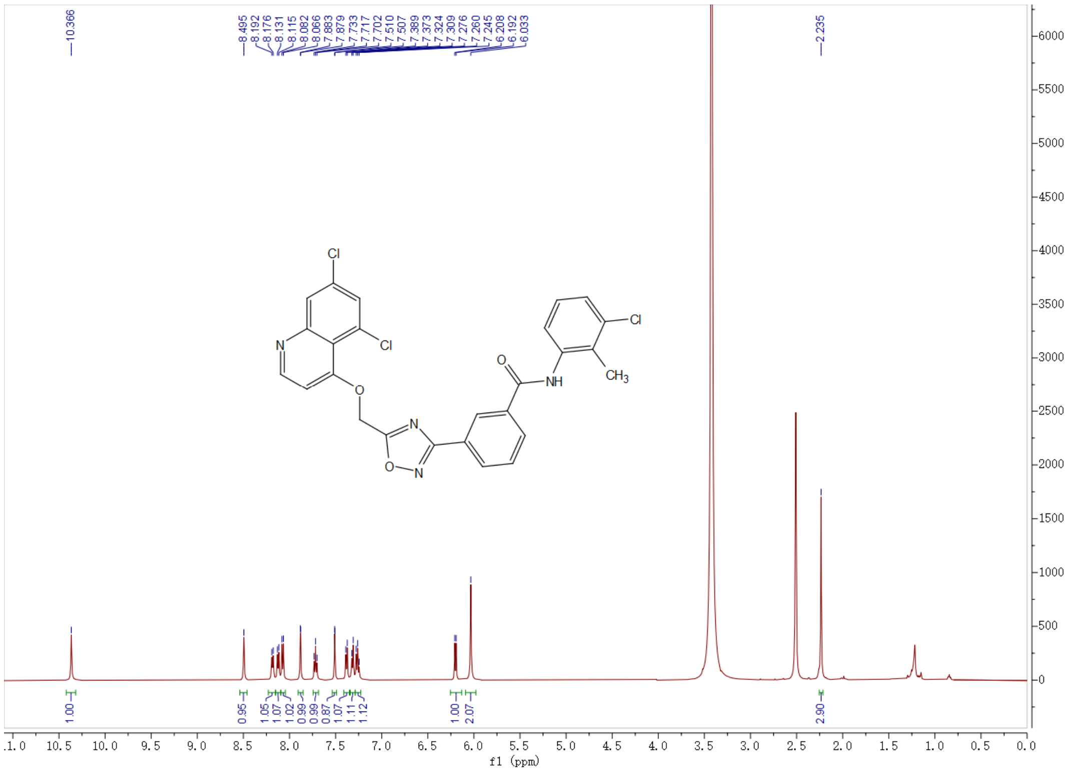

Figure S15. ^{1}H NMR spectra of **13o.**

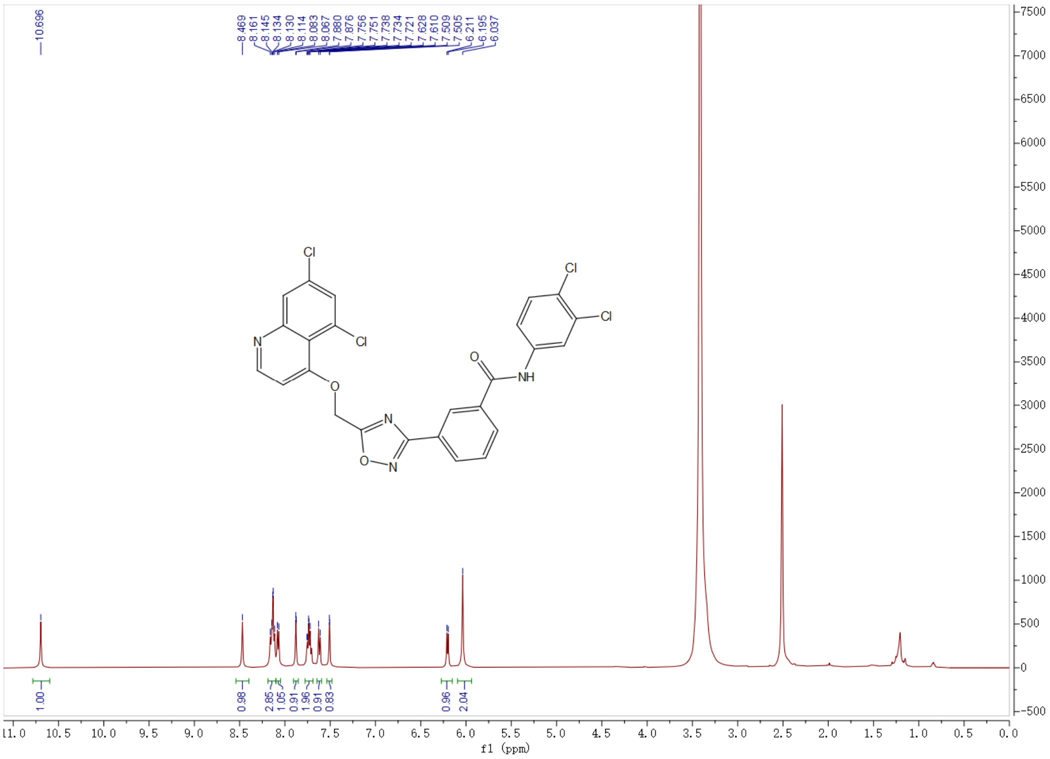

Figure S16. ^{1}H NMR spectra of **13p.**

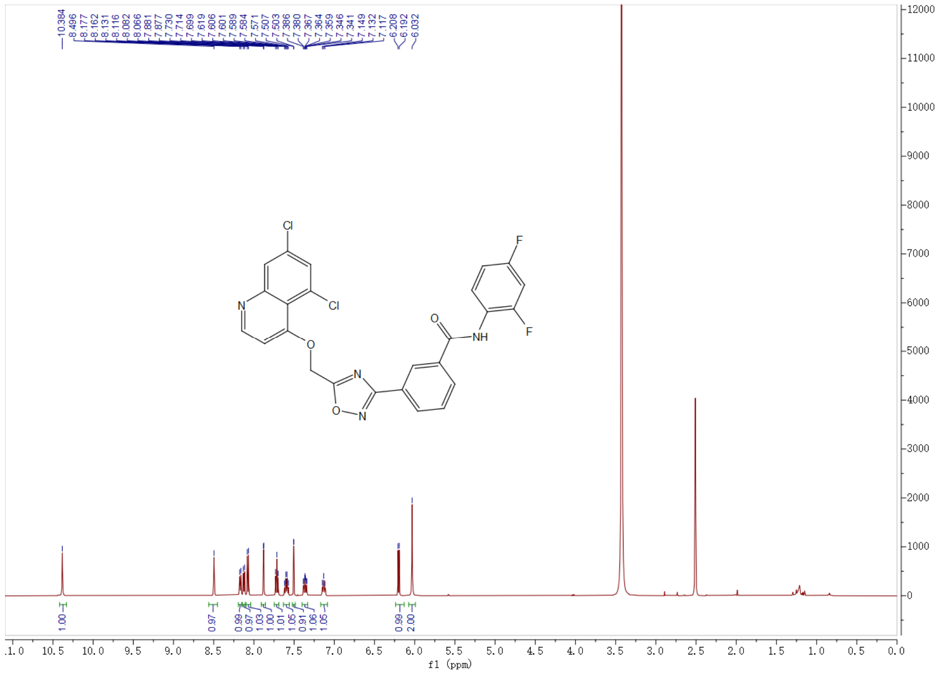

Figure S17. ^{1}H NMR spectra of **13q.**

2. ^{13}C NMR spectra of 13a~13q

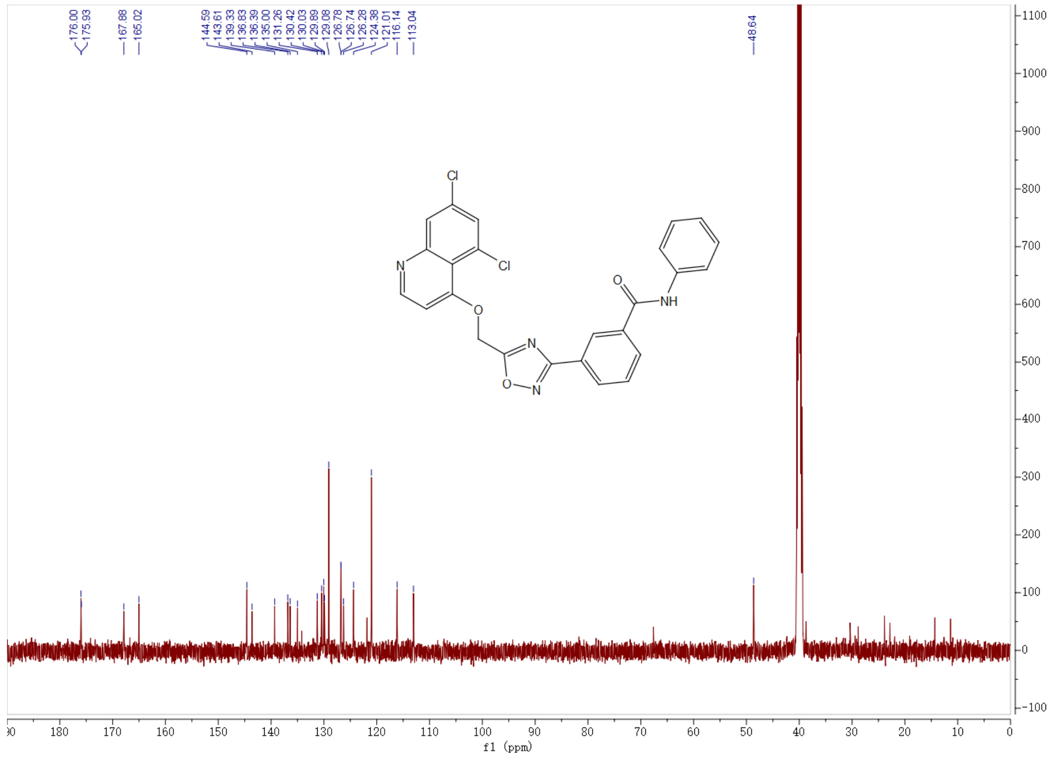

Figure S18. ^{13}C NMR spectra of **13a.**

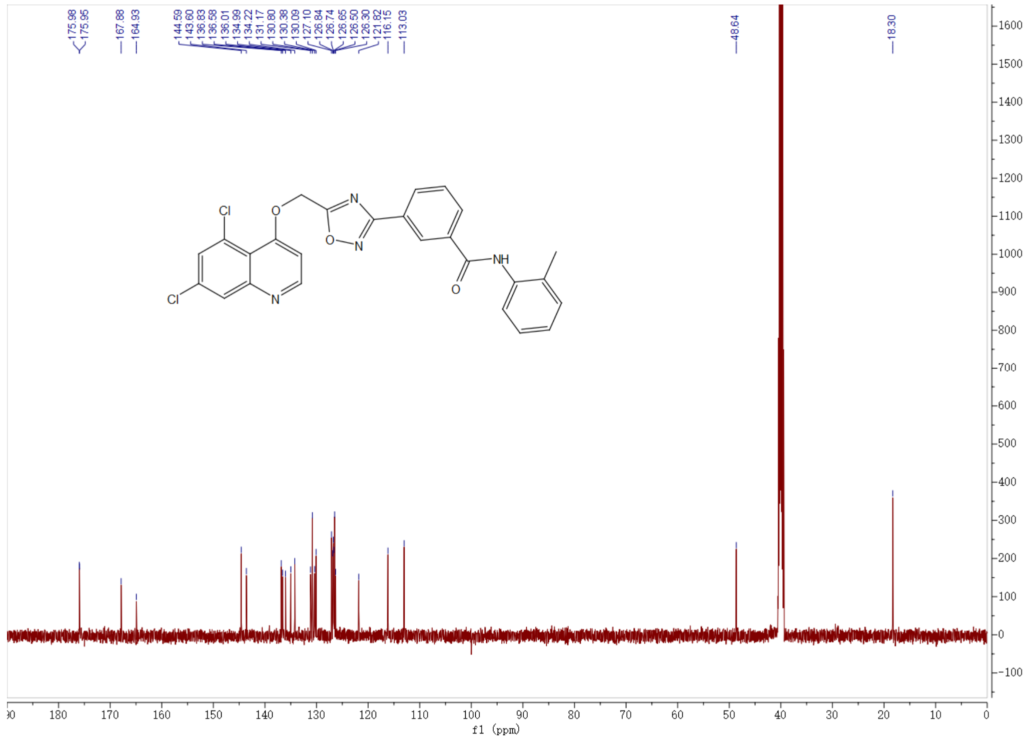

Figure S19. ^{13}C NMR spectra of **13b.**

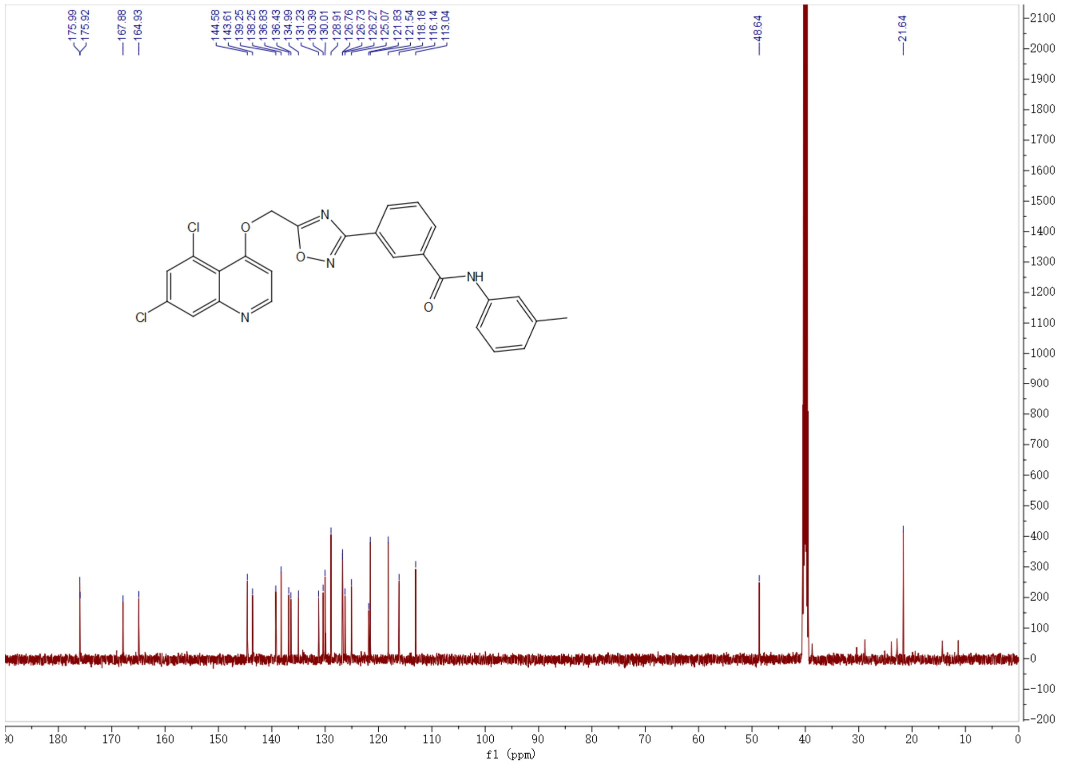

Figure S20. ^{13}C NMR spectra of **13c.**

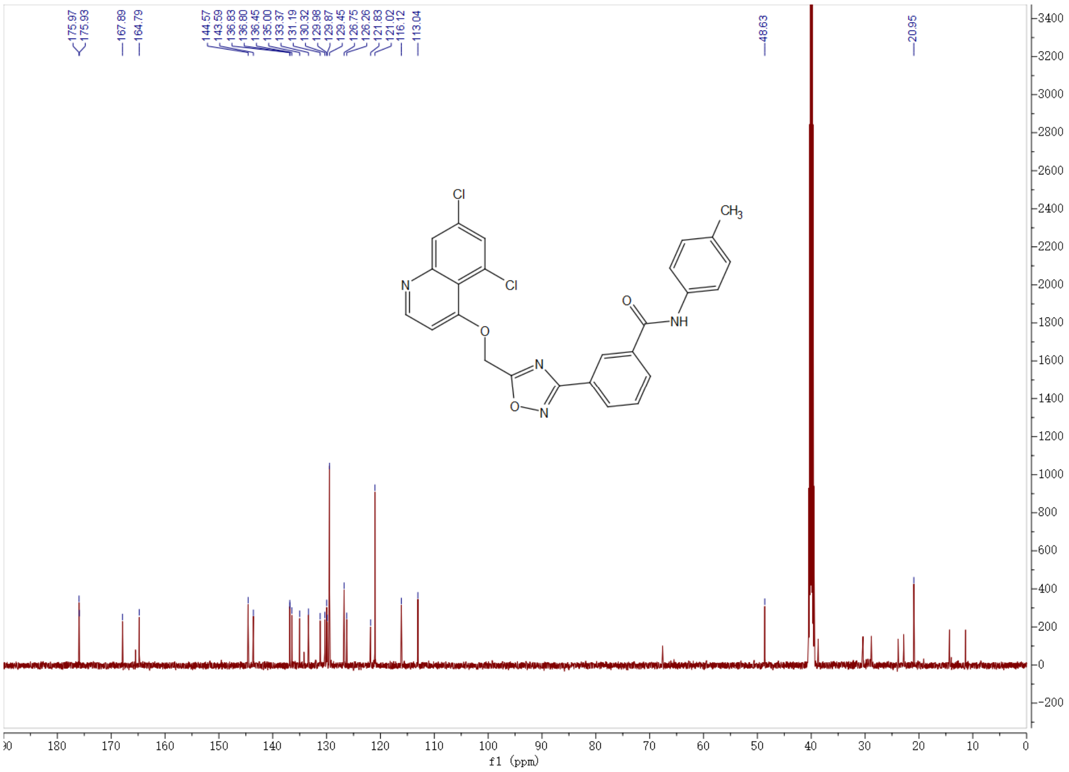

Figure S21. ^{13}C NMR spectra of **13d.**

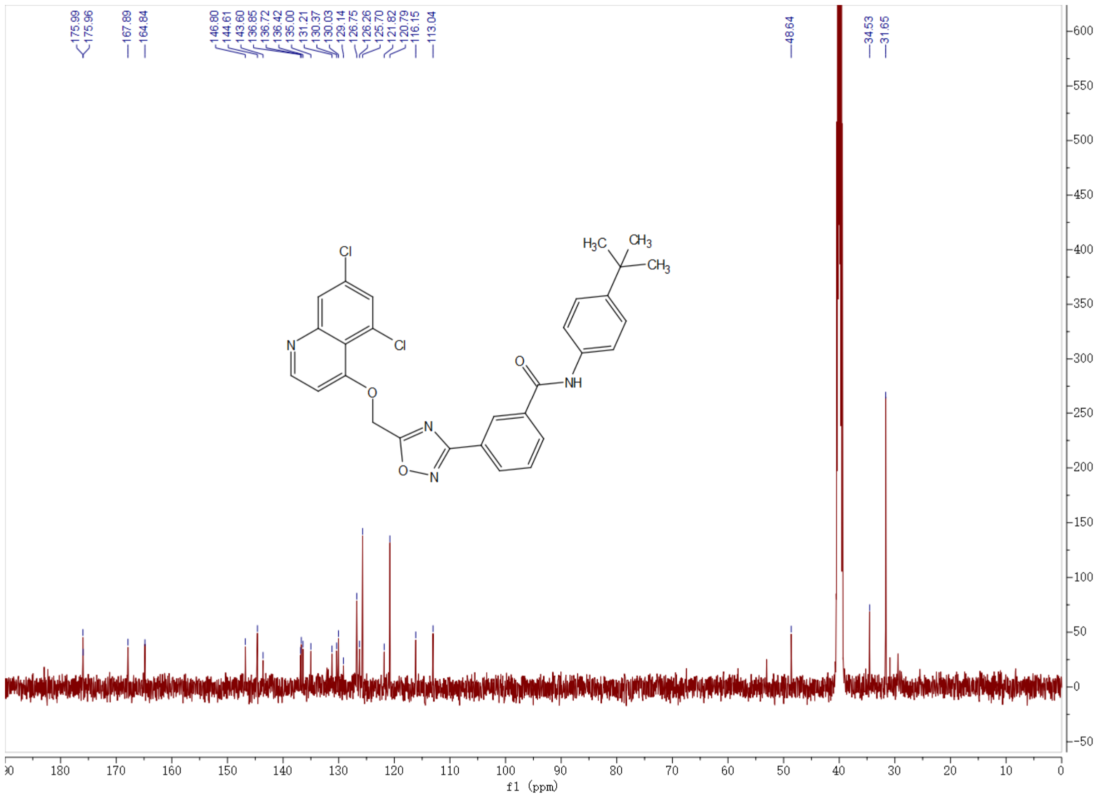

Figure S22. ^{13}C NMR spectra of **13e.**

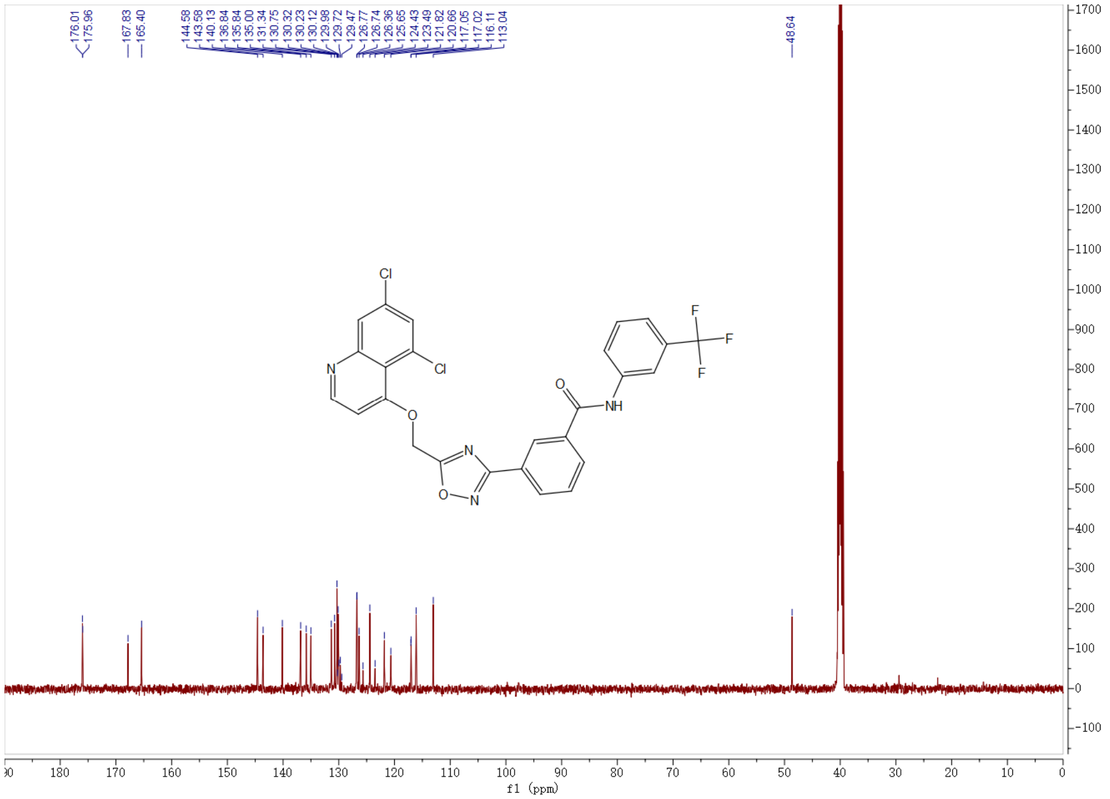

Figure S23. ^{13}C NMR spectra of **13f.**

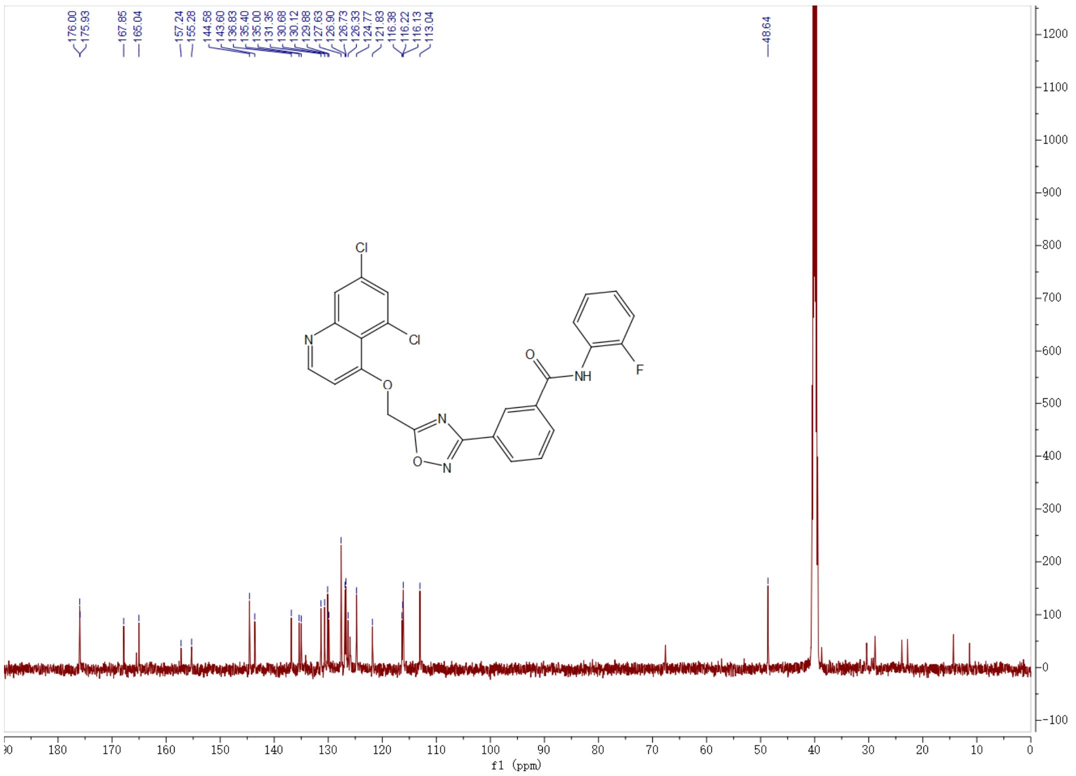

Figure S24. ^{13}C NMR spectra of **13g.**

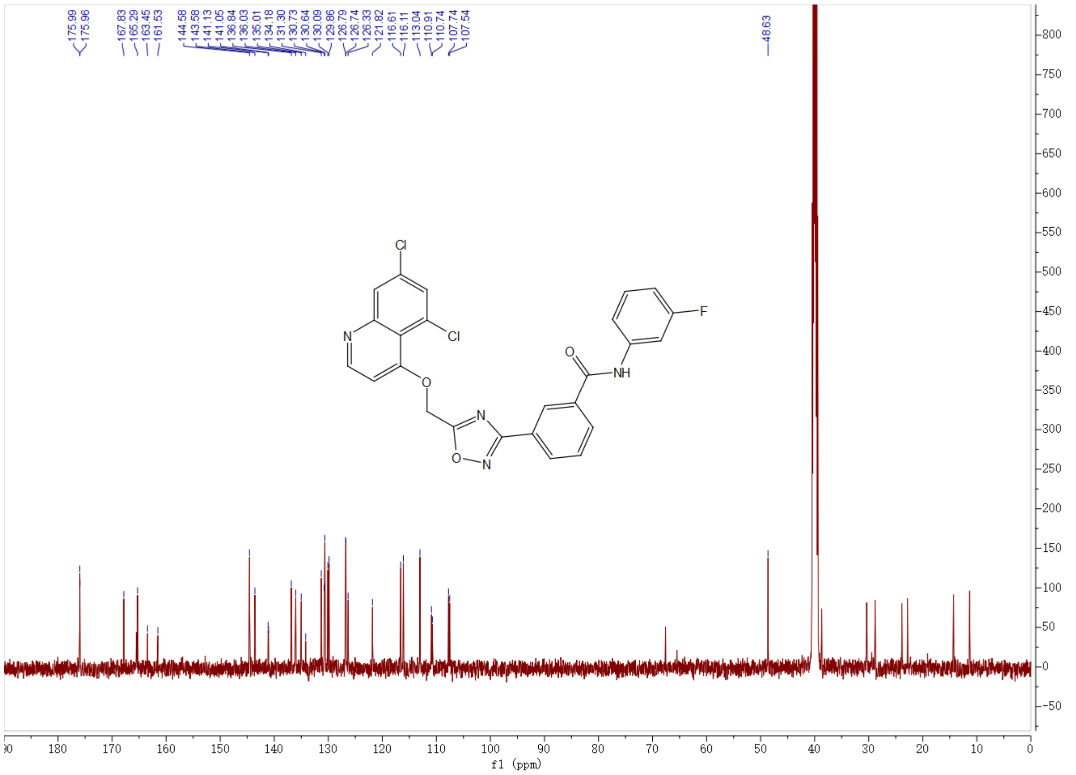

Figure S25. ^{13}C NMR spectra of **13h.**

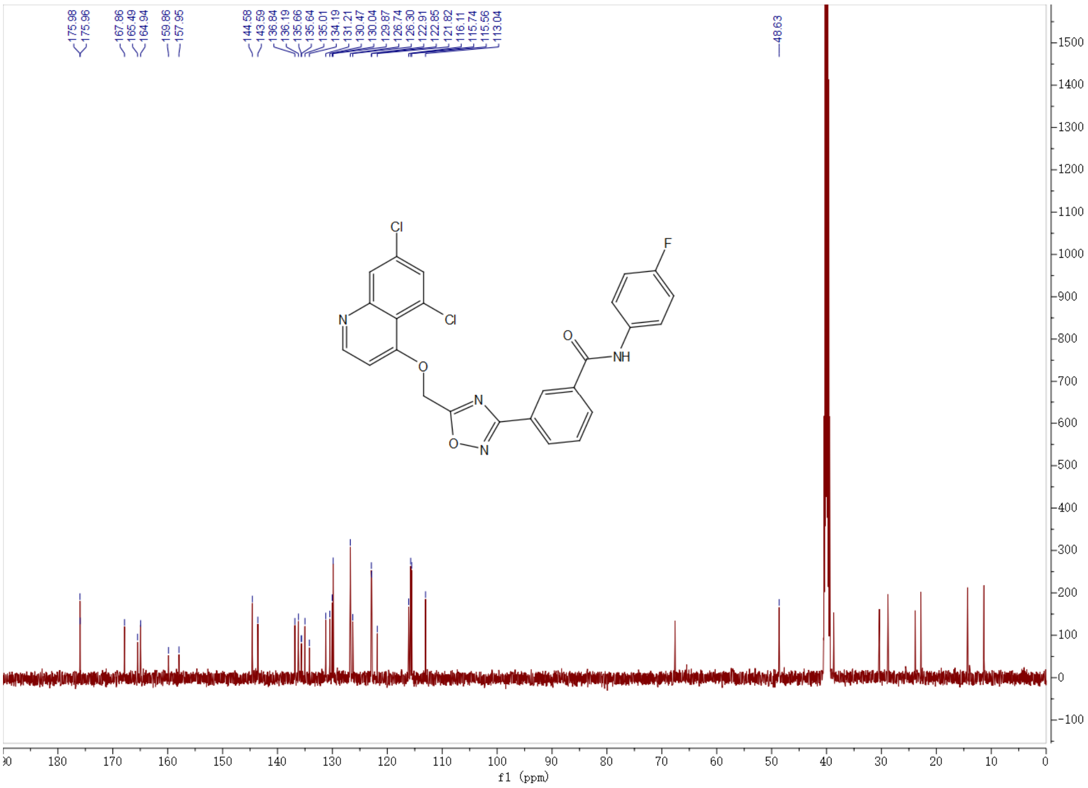

Figure S26. ^{13}C NMR spectra of **13i.**

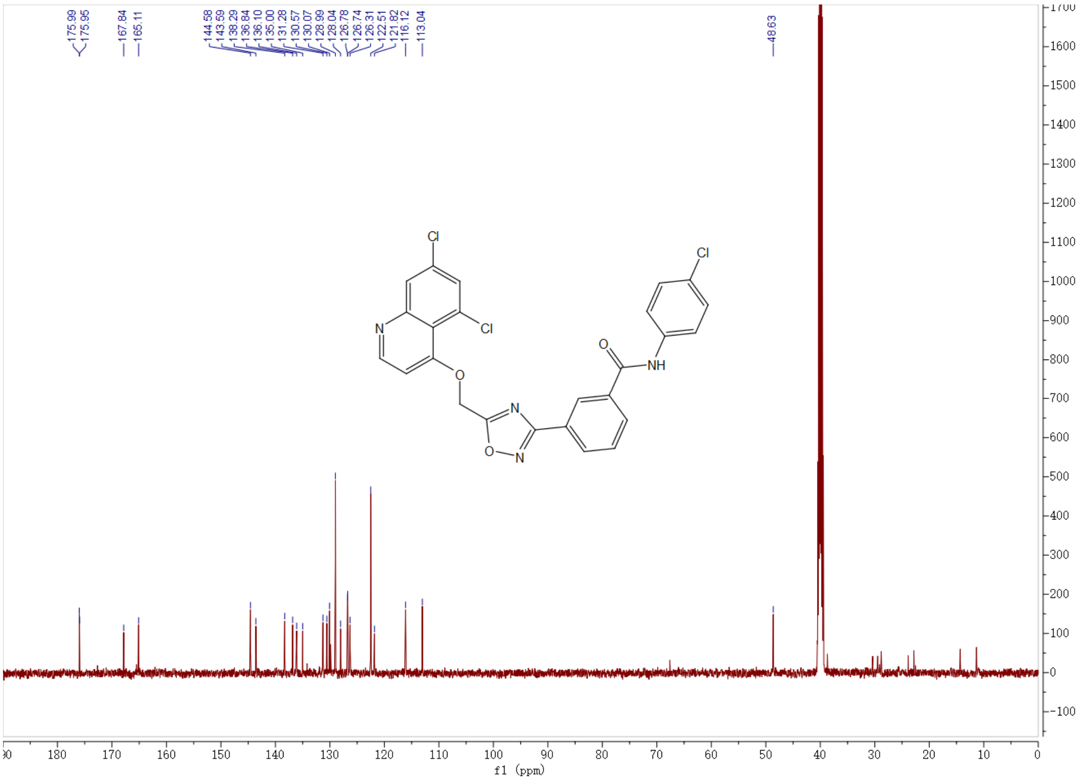

Figure S27. ^{13}C NMR spectra of **13j.**

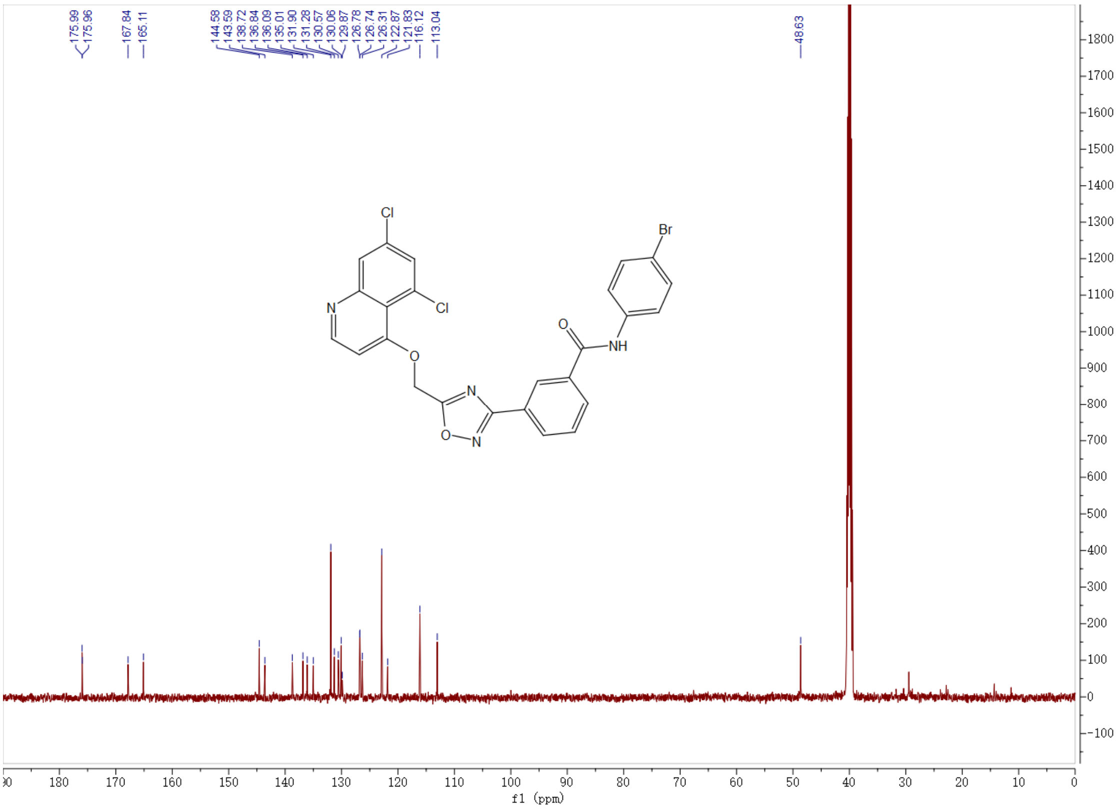

Figure S28. ^{13}C NMR spectra of **13k.**

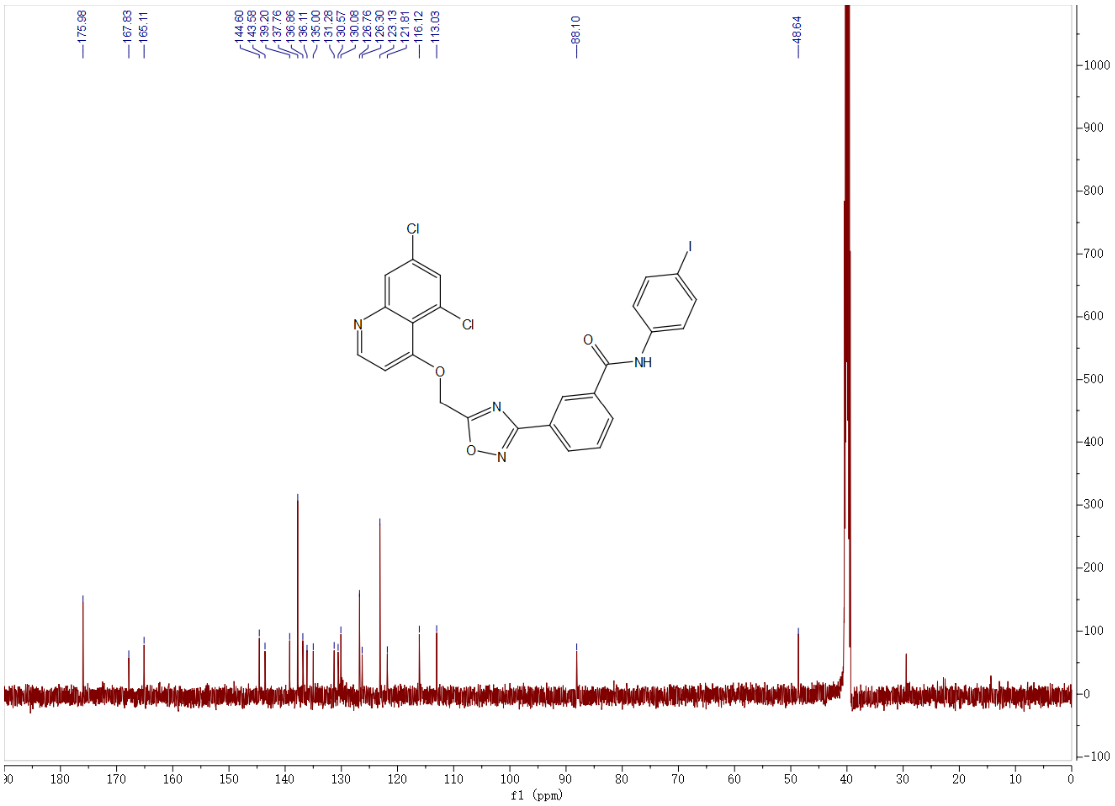

Figure S29. ^{13}C NMR spectra of **13l.**

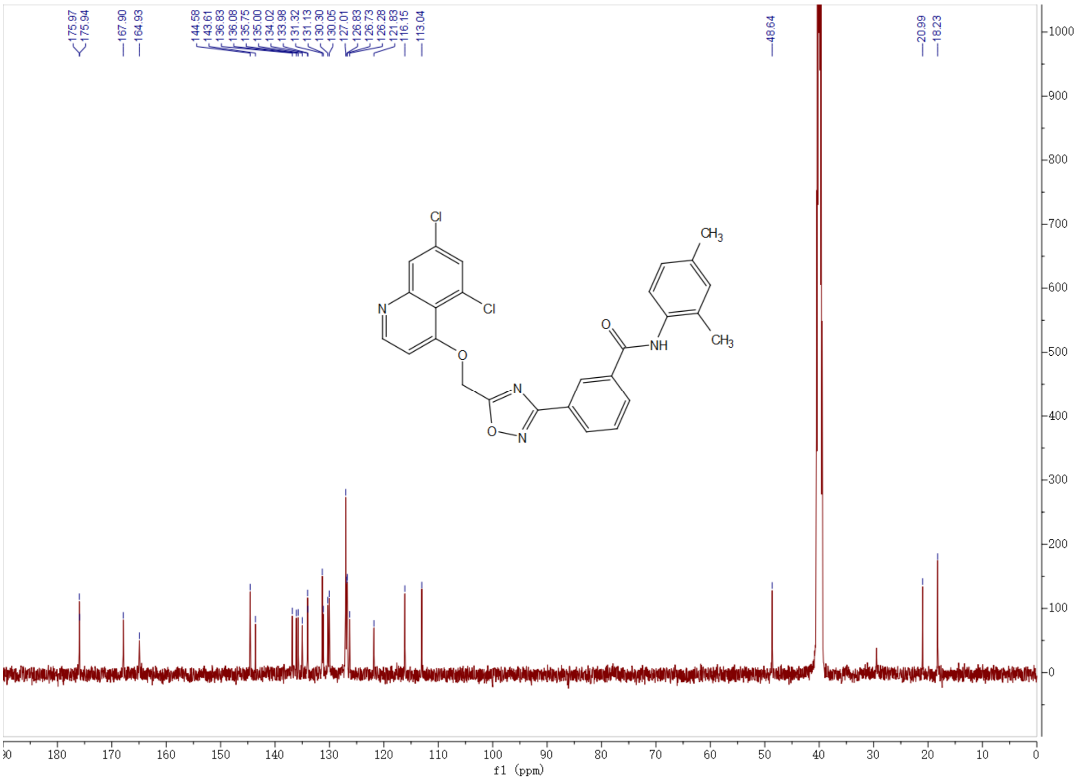

Figure S30. ^{13}C NMR spectra of **13m.**

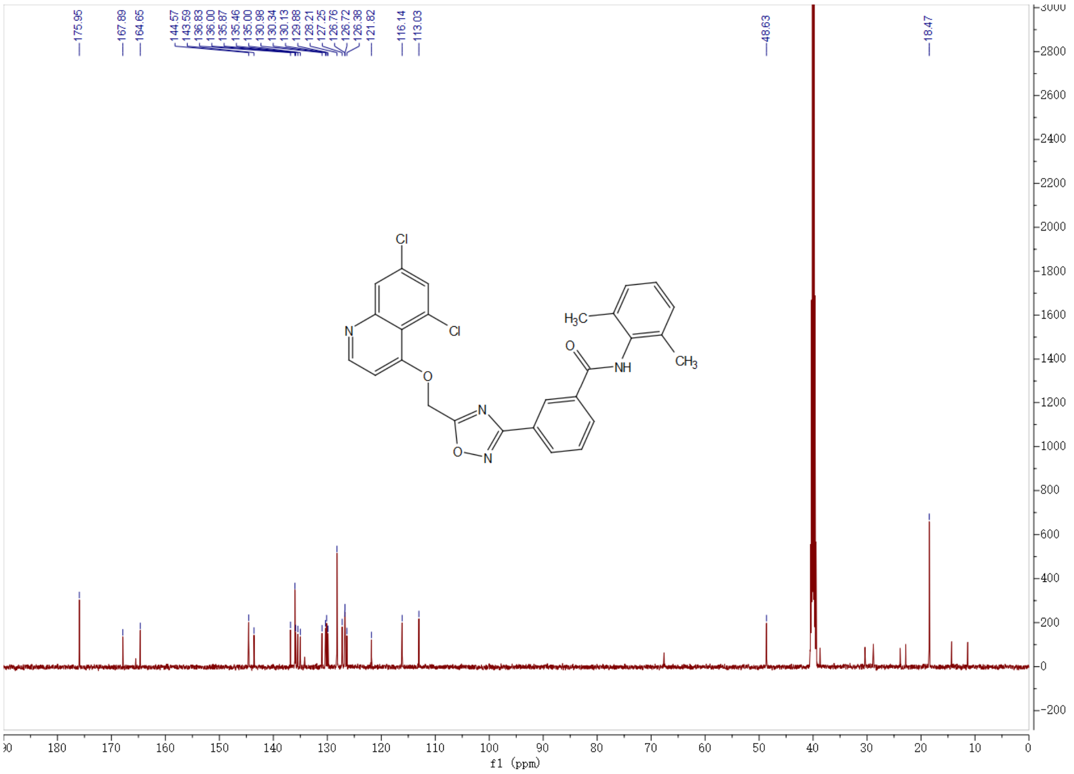

Figure S31. ^{13}C NMR spectra of **13n.**

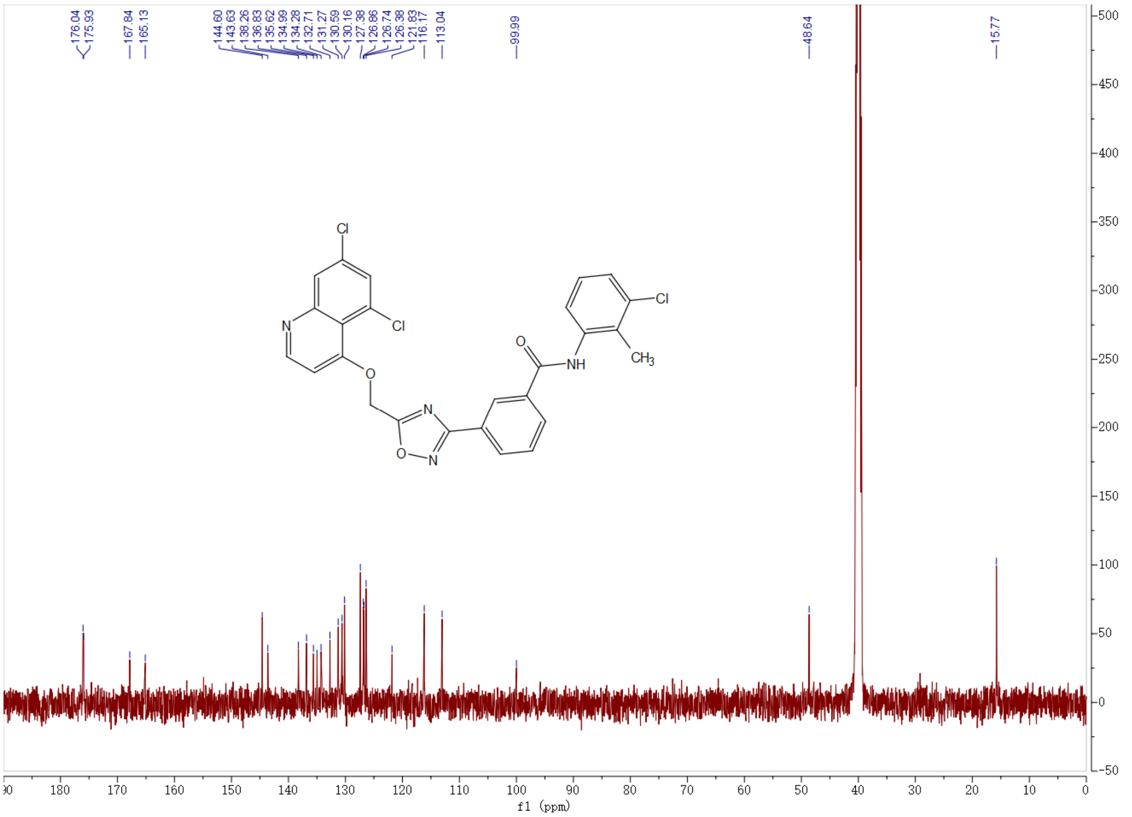

Figure S32. ^{13}C NMR spectra of **13o.**

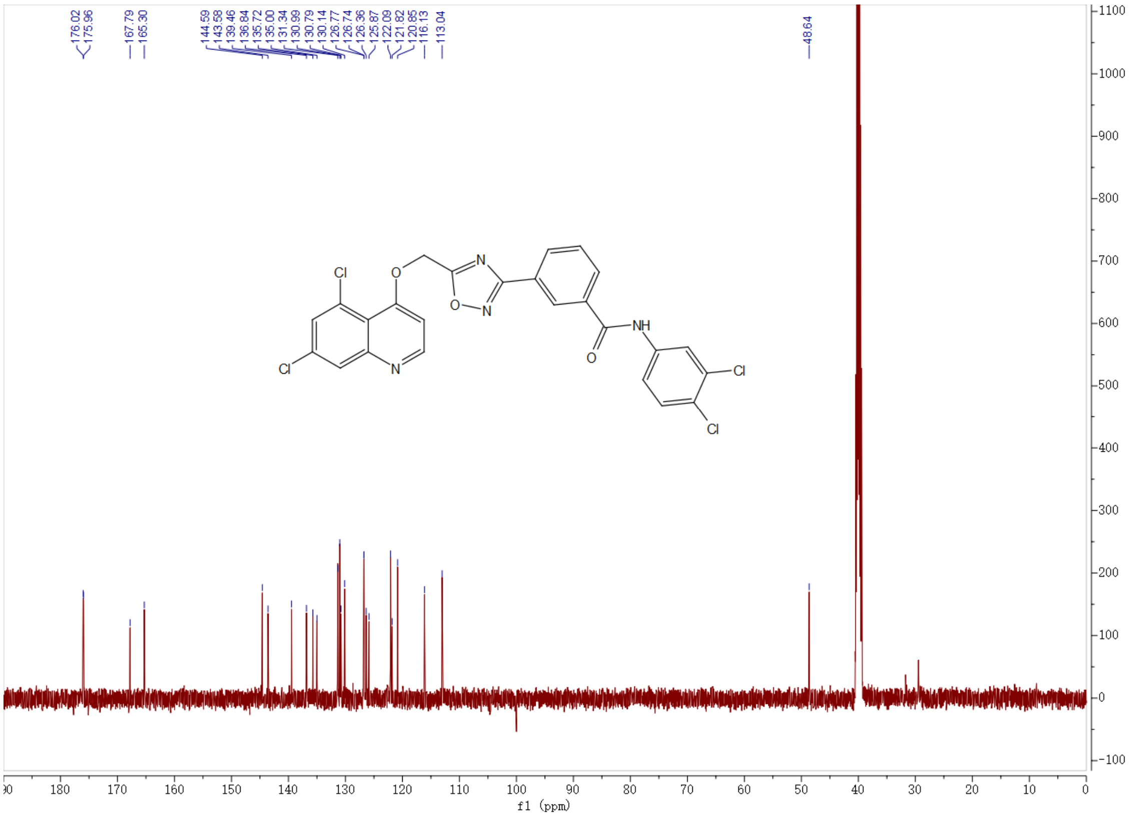

Figure S33. ^{13}C NMR spectra of **13p.**

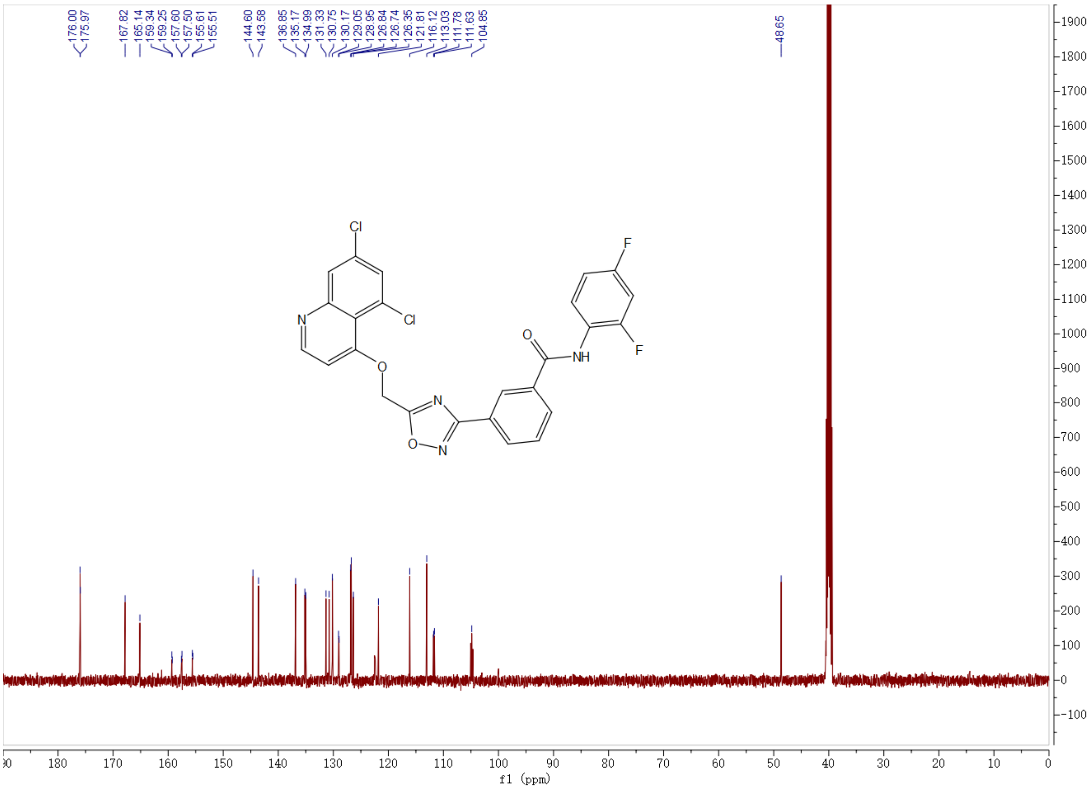

Figure S34. ^{13}C NMR spectra of **13q.**

3. ESI-HRMS spectra of **13a~13q**

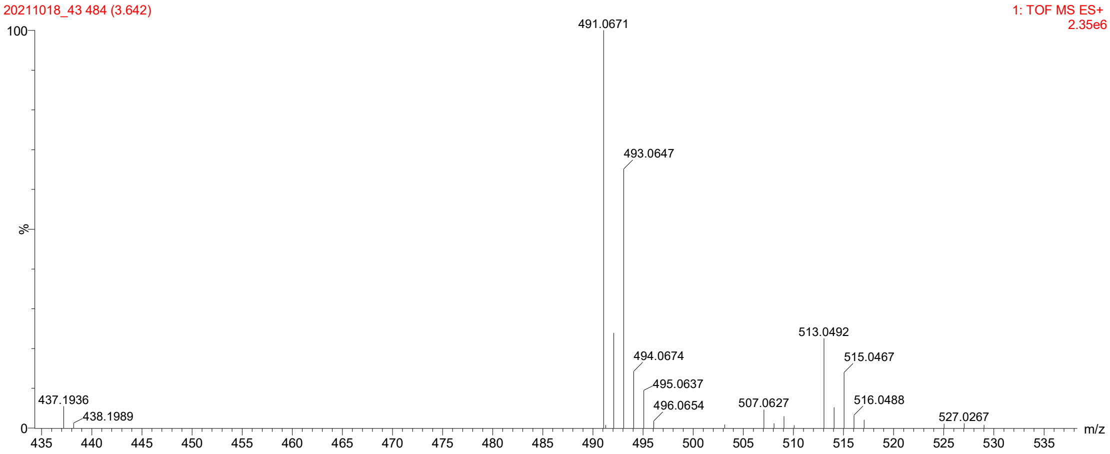

Figure S35. ESI-HRMS spectra of **13a.**

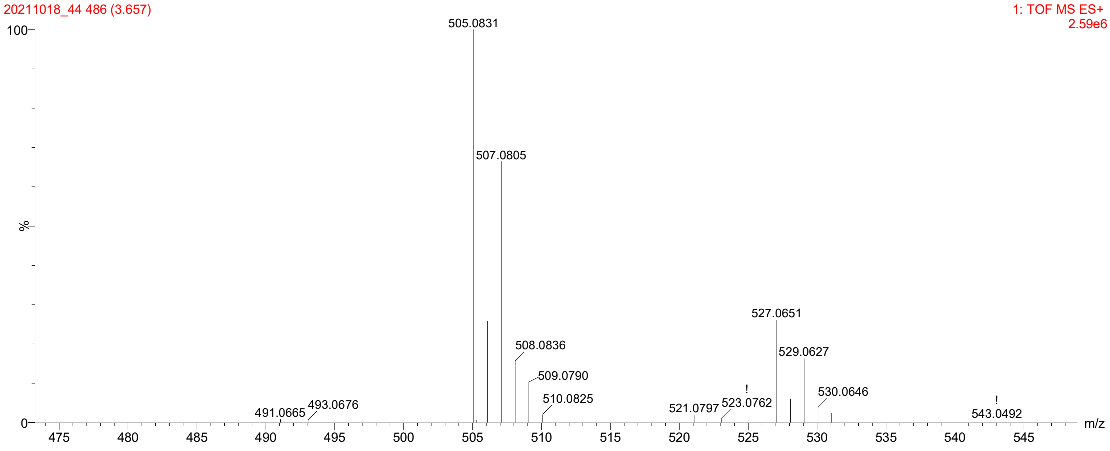

Figure S36. ESI-HRMS spectra of **13b.**

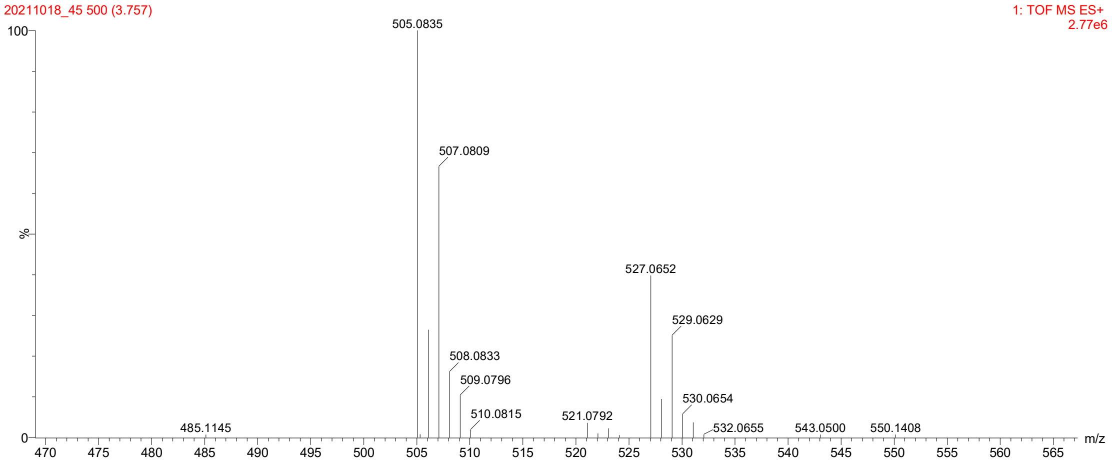

Figure S37. ESI-HRMS spectra of **13c.**

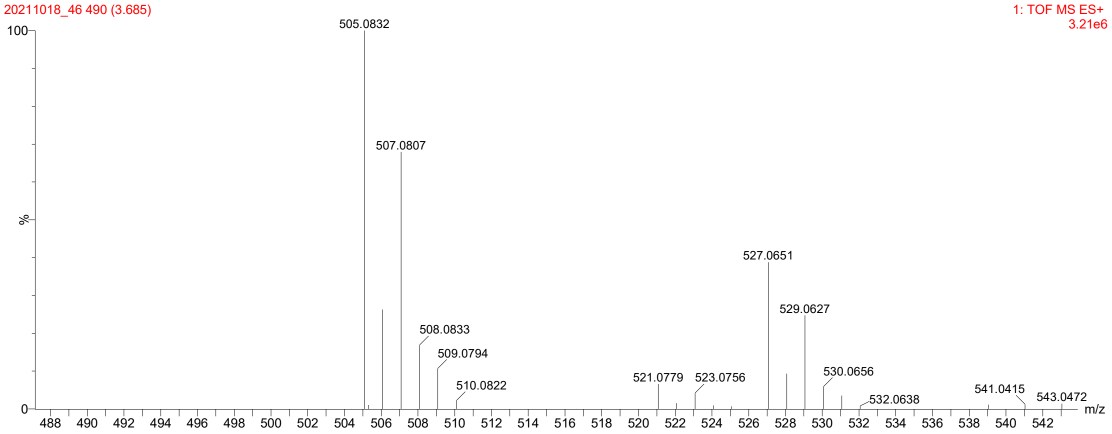

Figure S38. ESI-HRMS spectra of **13d.**

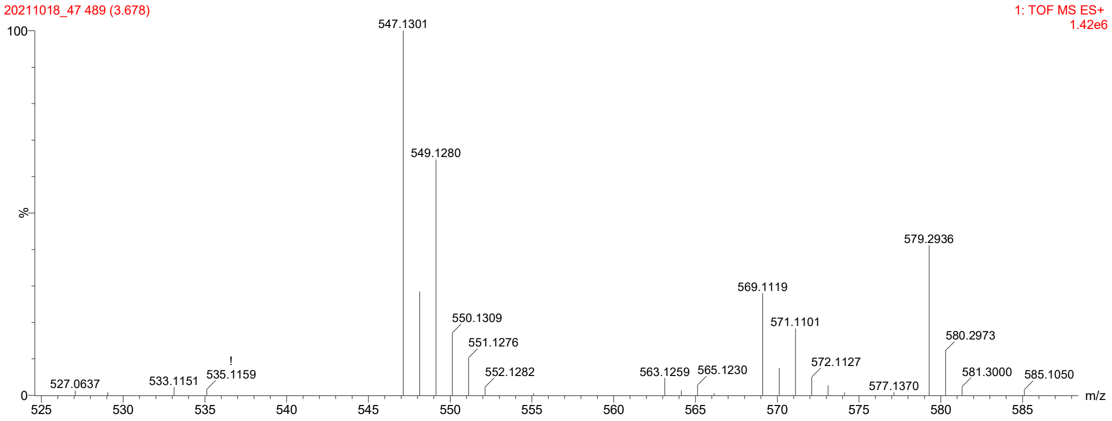

Figure S39. ESI-HRMS spectra of **13e.**

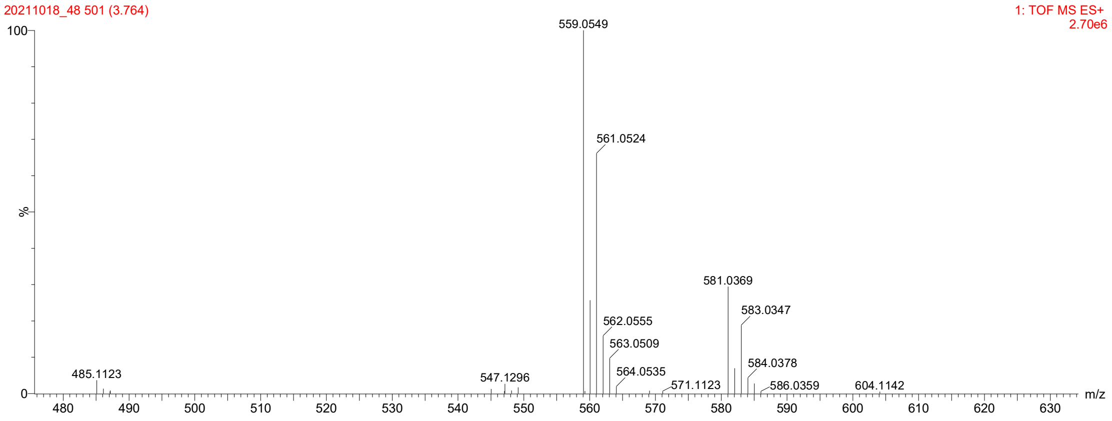

Figure S40. ESI-HRMS spectra of **13f.**

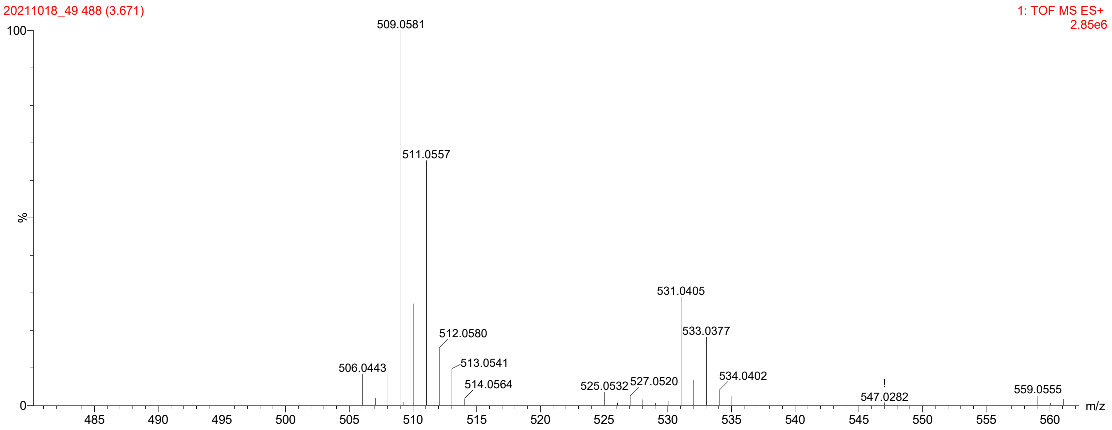

Figure S41. ESI-HRMS spectra of **13g.**

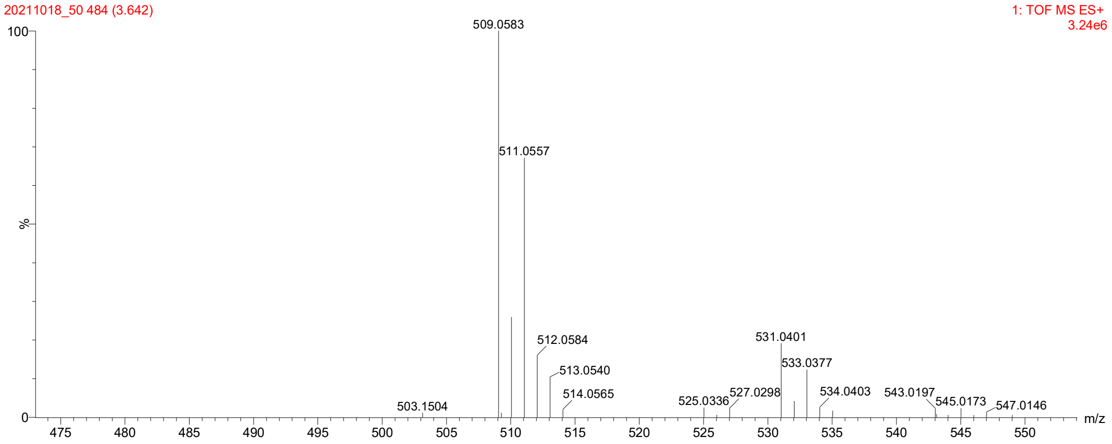

Figure S42. ESI-HRMS spectra of **13h.**

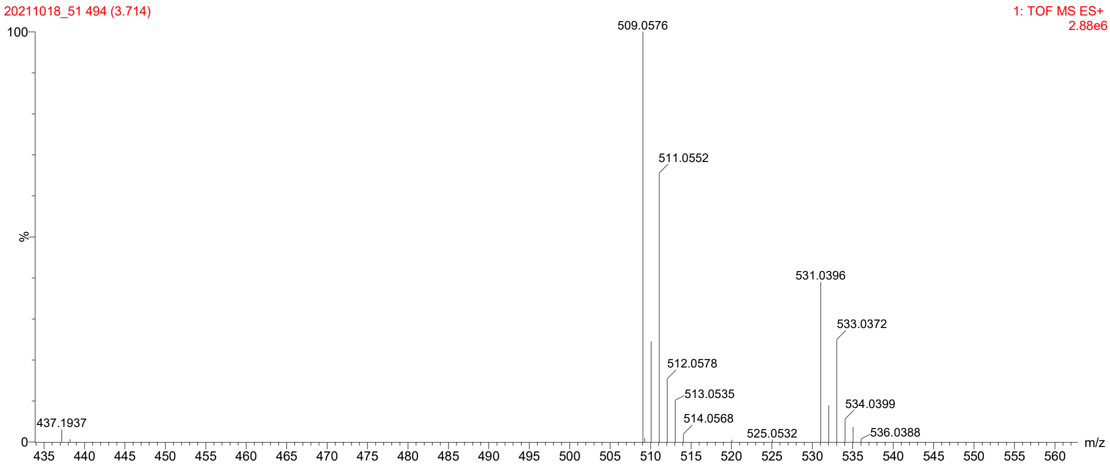

Figure S43. ESI-HRMS spectra of **13i.**

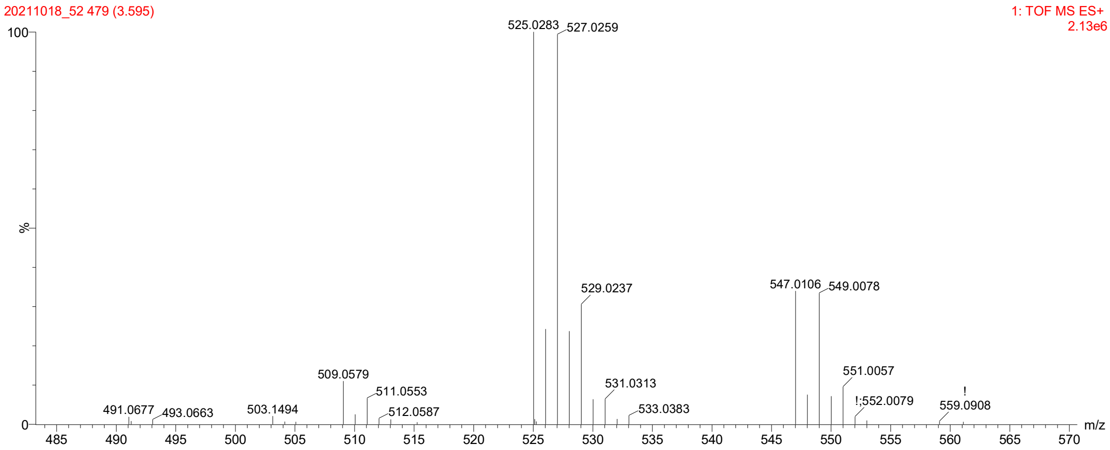

Figure S44. ESI-HRMS spectra of **13j.**

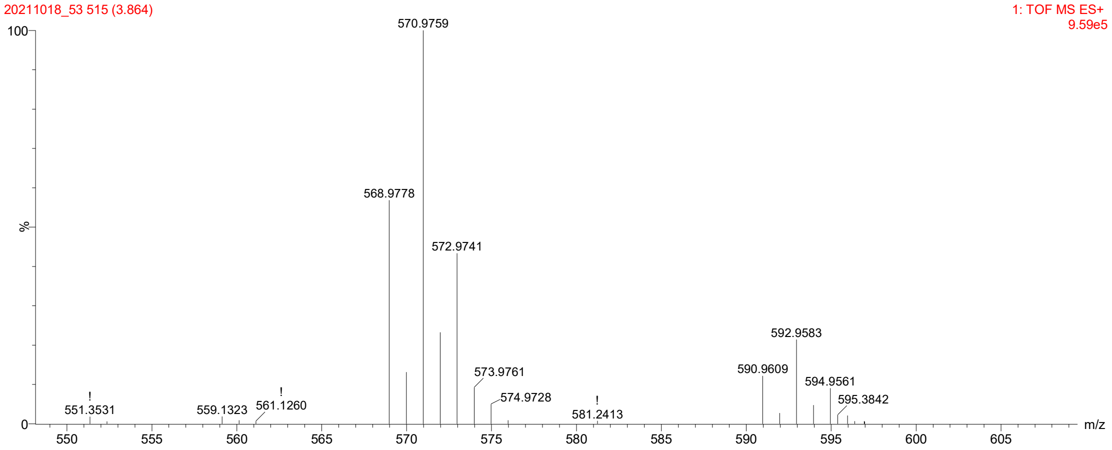

Figure S45. ESI-HRMS spectra of **13k.**

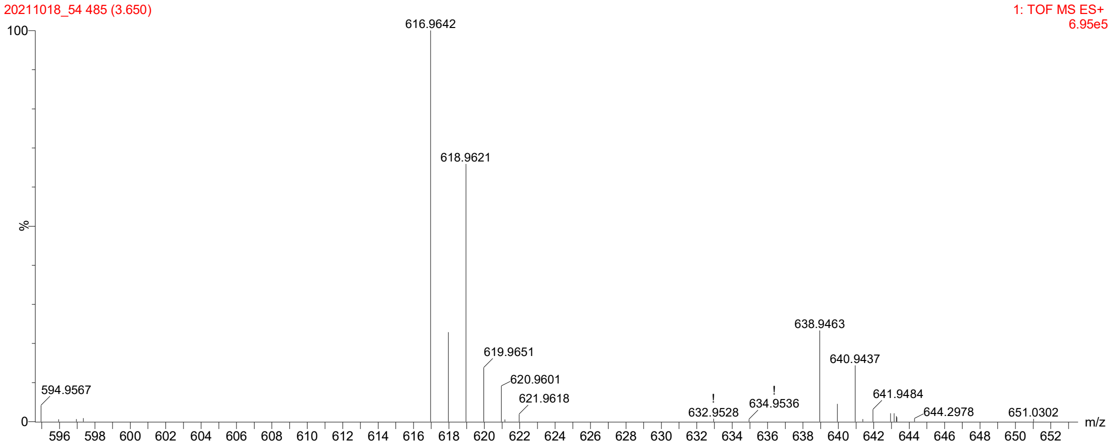

Figure S46. ESI-HRMS spectra of **13l.**

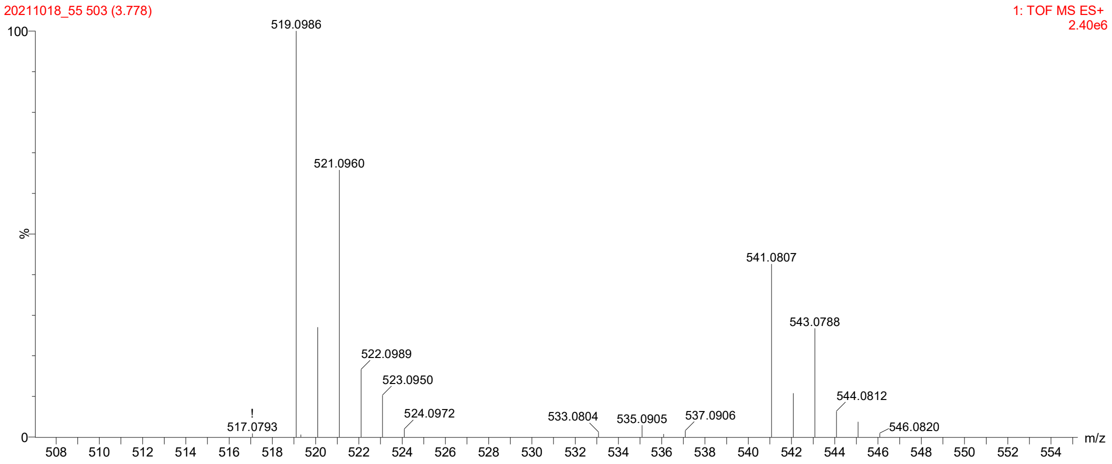

Figure S47. ESI-HRMS spectra of **13m.**

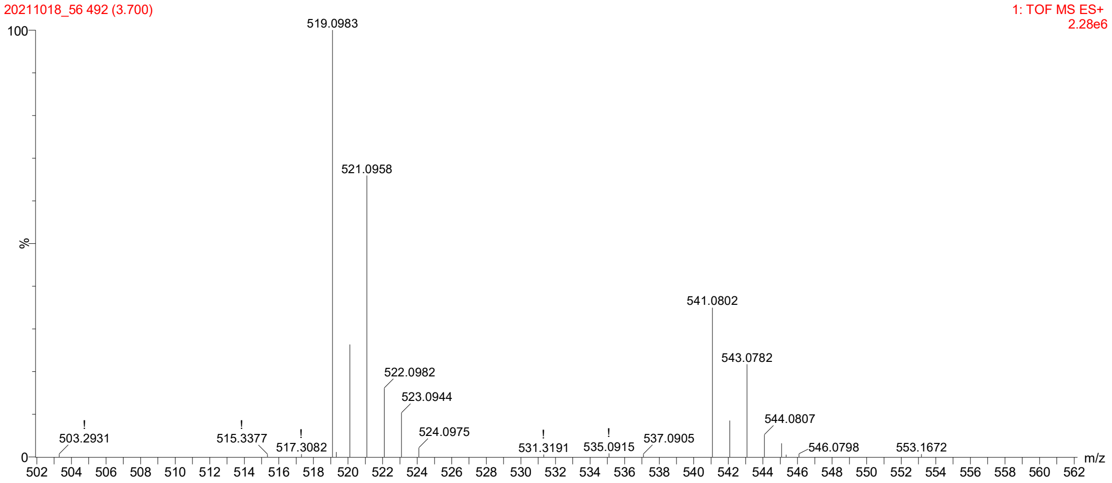

Figure S48. ESI-HRMS spectra of **13n.**

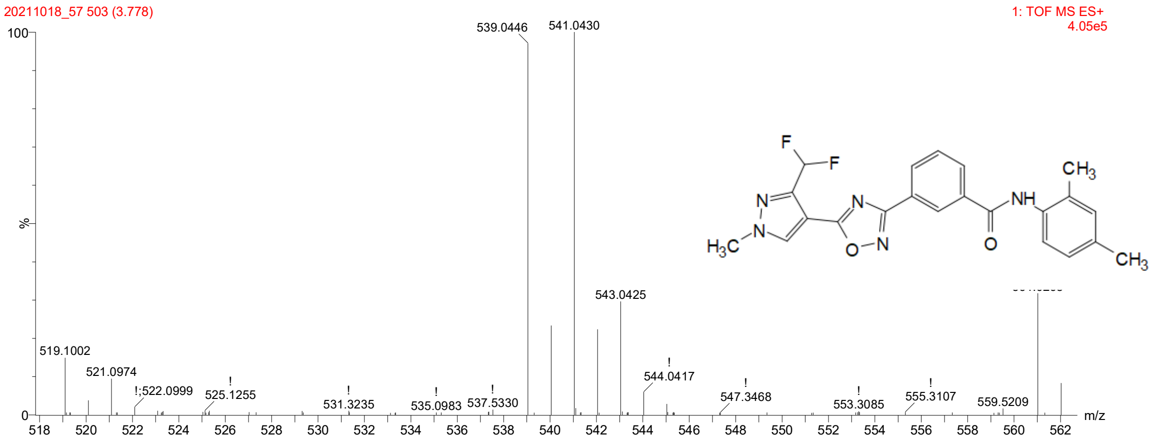

Figure S49. ESI-HRMS spectra of **13o.**

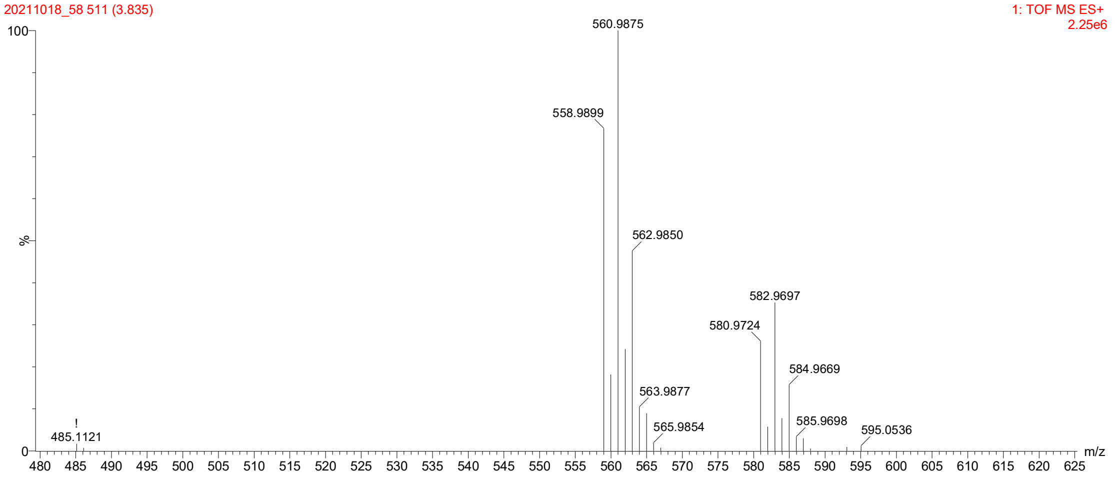

Figure S50. ESI-HRMS spectra of **13p.**

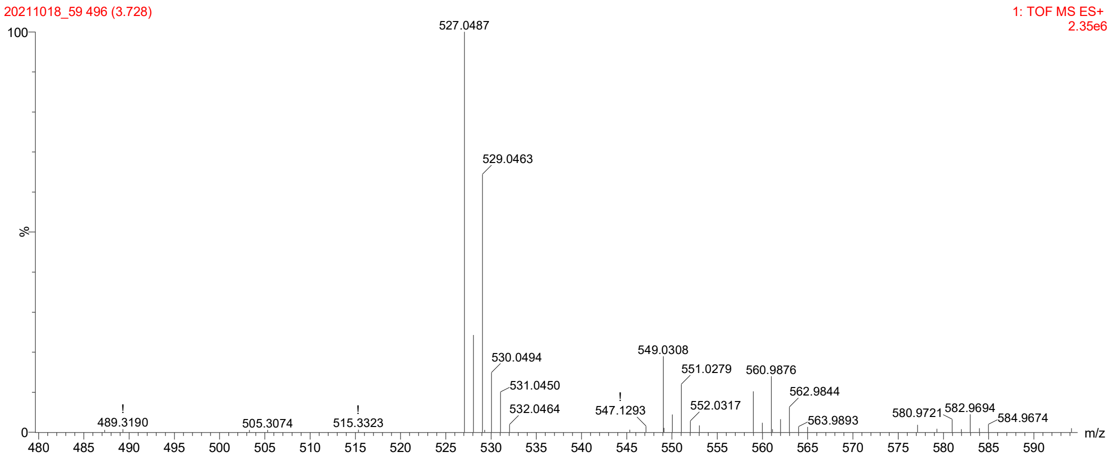

Figure S51. ESI-HRMS spectra of **13q.**
